# Binding Site Switch by Dispersion Interactions: Rotational Signatures of Fenchone–Phenol and Fenchone–Benzene Complexes

**DOI:** 10.1002/chem.202001713

**Published:** 2020-08-13

**Authors:** Ecaterina Burevschi, Elena R. Alonso, M. Eugenia Sanz

**Affiliations:** ^1^ Department of Chemistry King's College London London SE1 1DB UK

**Keywords:** density functional calculations, molecular recognition, noncovalent interactions, odorants, rotational spectroscopy

## Abstract

Non‐covalent interactions between molecules determine molecular recognition and the outcome of chemical and biological processes. Characterising how non‐covalent interactions influence binding preferences is of crucial importance in advancing our understanding of these events. Here, we analyse the interactions involved in smell and specifically the effect of changing the balance between hydrogen‐bonding and dispersion interactions by examining the complexes of the common odorant fenchone with phenol and benzene, mimics of tyrosine and phenylalanine residues, respectively. Using rotational spectroscopy and quantum chemistry, two isomers of each complex have been identified. Our results show that the increased weight of dispersion interactions in these complexes changes the preferred binding site in fenchone and sets the basis for a better understanding of the effect of different residues in molecular recognition and binding events.

## Graphical abstract

**Time to switch**! The interplay between hydrogen‐bonding and dispersion interactions has been investigated though the study of complexes of the odorant fenchone with phenol and benzene, mimics of tyrosine and phenylalanine residues, respectively. Two isomers of each complex have been characterised by rotational spectroscopy (see figure). The increased contribution of dispersion interactions in these complexes is responsible for a change in the preferred binding site in fenchone.

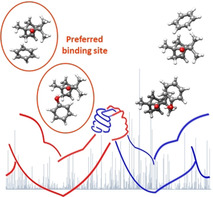

## Introduction

Intermolecular non‐covalent interactions play a subtle but decisive role in nature and occur in numerous chemical and biological processes.^1^ They are responsible for the existence of the liquid phase, determine the structures of biomacromolecules such as DNA and RNA, and are essential for molecular recognition in antigen–antibody, ligand–protein and odorant–receptor interactions, to name but a few.^2^, ^3^, ^4^, ^5^ The effects of non‐covalent interactions rest on the balance between the different forces (hydrogen bonding, dipole–dipole, dispersion) acting on a molecular system, which can be quite difficult to predict as the size of the system increases. It is thus important to have suitable small molecular models that can serve as test beds for different types of intermolecular interactions and improve their theoretical description.

In the sensory system, olfaction is responsible for the detection of odours by means of a chemoreception mechanism in which intermolecular interactions play one of the main roles. It is accepted that a combinatorial scheme is in place to enable the perception of thousands of smells with a few hundred odorant receptors, in which a given odorant can bind to and activate different receptors and one receptor can bind to different odorants.^6^ Olfactory receptors are G‐protein‐coupled receptors (GPCR) composed of seven transmembrane domain proteins.^7^ Therefore an odorant will participate in molecular recognition events involving different sets of amino acid residues. Further knowledge of the interactions contributing to odorant binding and recognition will help determine their relative importance and help modulate how interactions influence each other.

The structures of odorant receptors and odorant binding sites have not been resolved,^8^ but modelling studies have predicted the existence of hydrogen bonding and hydrophobic (dispersive) interactions mediated by the amino acid residues of serine, tyrosine and phenylalanine, among others.^9^, ^10^, ^11^, ^12^ We have started investigating the interactions of common odorants with mimics of amino acid residues and recently reported the interactions of the odorant fenchone, present in natural essential oils^13^, ^14^ and widely used in household products, with ethanol,^15^ chosen as a mimic of the side chain of serine. Fenchone has a rigid structure,^16^ which makes it easier to analyse its interactions with different partners as its conformational flexibility does not need to be considered. We found that O−H⋅⋅⋅O hydrogen bonding is the primary interaction in fenchone–ethanol complexes, but secondary interactions (including C−H⋅⋅⋅O hydrogen bonds and dispersion interactions) play a key role in determining the preferred arrangements of the monomers.

Here, we present the investigation of the complexes of fenchone with phenol and benzene, to probe the effect of changing the balance between intermolecular forces on the configurations of the aggregates. Phenol, like ethanol, has a hydroxy group that can act as hydrogen‐bond donor or acceptor, but in contrast to ethanol, it is bonded to an aromatic benzene ring rather than a short aliphatic chain. The presence of an aromatic ring introduces the possibility of forming C−H⋅⋅⋅π interactions and changes the potential dispersion interactions. Benzene shares the presence of an aromatic ring with phenol but lacks an ‐OH group, which allows us to determine the influence of not having a strong O−H⋅⋅⋅O hydrogen bond on structural preferences. Phenol and benzene are models for tyrosine and phenylalanine residues, respectively. By studying their complexes, and comparing them with those of fenchone with ethanol^15^ and water,^17^ we will obtain information on the interplay between the different hydrogen‐bonding and dispersion interactions.

We conducted our investigation using broadband rotational spectroscopy in a supersonic jet,^18^ a technique that allows interrogation of the isolated complexes free from solvent and lattice effects. Rotational spectroscopy is ideal for the investigation of intra‐ and intermolecular interactions because it provides exquisite structural resolution, which enables unequivocal identification of isomers and conformers simultaneously present in a sample, and estimation of their relative abundances. Broadband rotational spectroscopy allows broadband spectral collection, typically covering several GHz at once, which is very helpful for finding spectral patterns. Over the last few years, broadband rotational spectroscopy has been successfully applied to the investigations of multiconformational molecules, weakly bound complexes and reactive species.^19^, ^20^, ^21^, ^22^, ^23^, ^24^

In this study, we identified two complexes of fenchone–phenol and two complexes of fenchone–benzene. The most abundant isomers of each complex display the same relative configuration of fenchone and phenol or benzene, even though there are significant differences in the interactions involved. Interestingly, the location of phenol and benzene in their respective lowest‐energy isomers maximises the dispersion forces. The identification of several isomers allows benchmarking of computational calculations and yields valuable information on the interplay of intermolecular forces.

## Results

### Fenchone–phenol

The rotational spectrum of fenchone–phenol shows very intense lines that were identified as arising from the fenchone monomer.^16^ Lines from bare phenol,^25^ phenol dimer^26^ and, due to the presence of residual water in our injection line, complexes of fenchone–H_2_O^17^ and phenol–H_2_O^27^, ^28^ were also observed. All the transitions belonging to the above species were removed, considerably reducing the spectral line density. Considering that the predicted isomers of fenchone–phenol (FPHE) were nearly prolate asymmetric rotors with a large electric dipole moment along the *a* principal inertial axis *μ_a_* (see Table 1 and Table S1 in the Supporting Information), we searched for the typical pattern of *a*‐type R‐branch transitions, eventually finding two series of lines separated approximately by the sum of the rotational constants *B*+*C*. The spectroscopic constants obtained from the fit^29^ of all the observed transitions (see Tables S2 and S3) to Watson's *A*‐reduced semi‐rigid rotor Hamiltonian in the *I* ^r^ representation^30^ are shown in Table 2. By comparing the experimental rotational constants with those predicted theoretically we unambiguously identified FPHE1 and FPHE2 as isomers 1 and 2.

**Table 1:** B3LYP‐D3BJ and MP2 spectroscopic parameters and relative energies for the five isomers of the fenchone–phenol complex.

Isomer	**1**		**2**		**3**		**4**		**5**	

	B3LYP‐ D3BJ^[a]^	MP2	B3LYP‐ D3BJ	MP2	B3LYP‐ D3BJ	MP2^[b]^	B3LYP‐ D3BJ	MP2	B3LYP‐ D3BJ	MP2
*A* ^[c]^ [MHz]	698.7	677.9	903.8	853.7	852.5	–	716.2	679.3	854.9	742.4
*B* [MHz]	294.7	325.0	212.2	229.0	211.2	–	266.6	299.5	215.4	278.5
*C* [MHz]	279.2	314.4	200.4	215.4	206.6	–	250.3	282.9	206.6	268.3
*μ_a_* [D]	−3.3	−2.8	−4.5	3.9	4.6	–	−4.0	−3.3	4.7	3.4
*μ_b_* [D]	1.5	−2.0	0.0	0.2	0.0	–	−1.4	−1.7	0.0	0.7
*μ_c_* [D]	−2.5	2.1	−2.4	2.5	2.3	–	−2.4	−2.2	−2.6	−2.9
Δ*E* ^[d]^ [cm^−1^]	0	0	200	918	233	–	253	570	213	774
Δ*E*_ZPE_^[e]^ [cm^−1^]	0	0	140	782	161	–	206	456	382	673
*D*_e_^[f]^ [kJ mol^−1^]	46.8	35.4	45.3	33.3	45.0	–	44.3	33.0	42.8	31.6
	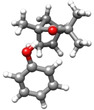		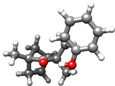		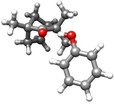		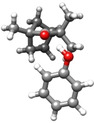		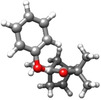	

[a] All calculations were performed using the 6‐311++G(d,p) basis set. [b] Isomer 3 converges to isomer 4 at the MP2 level of theory. [c] *A*, *B* and *C* are the rotational constants. *μ_a_*, *μ_b_* and *μ_c_* are the electric dipole moment components. [d] Relative electronic energies. [e] Relative electronic energies including the zero‐point correction. [f] Dissociation energies.

**Table 2:** Experimental spectroscopic constants of the observed isomers of fenchone–phenol.

	FPHE1	FPHE2
*A* ^[a]^ [MHz]	698.07125(67)^[b]^	910.23133(58)
*B* [MHz]	287.602617(99)	206.090858(73)
*C* [MHz]	272.41326(11)	192.996827(81)
Δ_*J*_ [kHz]	0.17753(35)	0.02927(16)
Δ_*JK*_ [kHz]	−0.3026(29)	−0.0643(23)
Δ_*K*_ [kHz]	0.306(66)	0.282(36)
*δ_J_* [kHz]	−0.01153(21)	–
*δ_K_* [kHz]	0.236(25)	–
*a*/*b*/*c* ^[c]^ [D]	s/m/m	s/–/m
*σ* ^[d]^ [kHz]	5.5	6.4
*N* ^[e]^	169	172

[a] *A*, *B* and *C* are the rotational constants. Δ_*J*_, Δ_*JK*_, Δ_*K*_, *δ_J_* and *δ_K_* are the quartic centrifugal distortion constants. [b] Standard error in parentheses in units of the last digit. [c] *a*, *b* and *c* are the type of transitions observed: strong, medium and weak. [d] *σ* is the r.m.s. deviation of the fit. [e] *N* is the number of fitted transitions.

Of the computational calculations (see Table 1 and Table S1 in the Supporting Information), the B3LYP‐D3BJ/6‐ 311++G(d,p) level predicts theoretical equilibrium rotational constants closest to the experimental values for the ground vibrational state (see Table S4). This was also observed for the fenchone–ethanol complex.^15^ There are considerable discrepancies between the B3LYP‐D3BJ and MP2 predictions, which are discussed in the following section in the context of competing intermolecular interactions.

Other predicted isomers of fenchone–phenol were searched for in the rotational spectrum. However, no patterns belonging to these species could be identified. Their non‐observation could be explained by collisional relaxation in the supersonic jet to lower‐energy isomers.^31^ We calculated the interconversion barriers of isomer 4 to 3, and isomer 3 to 2 through relaxed scans of the corresponding dihedral angles at the B3LYP‐D3BJ level of theory. In both cases, the barrier heights are well below 400 cm^−1^ (see Figure S1 in the Supporting Information), and thus compatible with the relaxation of isomers 3 and 4 in the supersonic expansion.

The relative intensities of the observed transitions, which in our instrument are directly proportional to the square of the corresponding dipole moment component, are consistent with the above assignment. The relative abundances of the observed isomers were estimated from measurements of *a*‐type transitions to be FPHE1/FPHE2=2:1 (see Figure S2 in the Supporting Information) by using the expression *N*_1_/*N*_2_=*I*_1_ *μ*_2_^2^/*I*_2_ *μ*_1_^2^, in which *N* is the number density of the corresponding isomer, *I* is the line intensity and *μ* is the corresponding dipole moment component. This value is in very good agreement with their dissociation energies and with their relative energies at the B3LYP‐D3BJ level. Calculations at the MP2 level, however, predict isomer 2 to have the highest energy.

### Fenchone–benzene

Similarly to fenchone–phenol, the rotational spectrum of fenchone–benzene (FBEN) is dominated by the lines of the fenchone monomer, with transitions from fenchone–H_2_O^17^ also being observed. Bare benzene does not have a dipole moment and therefore does not contribute to the spectrum. However, transitions arising from the benzene–H_2_O complex^32^, ^33^ are present. Once these transitions were removed, two sets of *c*‐type *J*+1_1*,J*−1_←*J*_0*,J*_ lines separated approximately by 2*B* were identified as corresponding to two isomers of fenchone–benzene, namely FBEN1 and FBEN2. The initial assignments were confirmed by subsequent measurement of more transitions. All the transitions (see Tables S5 and S6 in the Supporting Information) were fitted to Watson's *A*‐reduced Hamiltonian in the *I* ^r^ representation^30^ to determine the experimental constants presented in Table 3. From a comparison of these constants with theoretical values (Table 4 and Table S7), FBEN1 and FBEN2 were unambiguously identified as isomers 1 and 2, respectively. Other predicted low‐energy isomers of fenchone–benzene were searched for in the spectrum but no transitions that could be assigned to them were found. As for the fenchone–phenol complexes, the B3LYP‐D3BJ method produces equilibrium structures closest to the experimental ones (see Table S4).

**Table 3:** Experimental spectroscopic constants of the observed isomers of fenchone–benzene.

	FBEN1	FBEN2
*A* ^[a]^ [MHz]	767.45155(14)^[b]^	805.56766(35)
*B* [MHz]	335.063751(64)	314.56244(16)
*C* [MHz]	316.145309(62)	289.98294(19)
Δ_*J*_ [kHz]	0.04390(27)	0.04085(71)
Δ_*JK*_ [kHz]	0.0222(14)	0.0536(61)
*δ_J_* [kHz]	0.00195(18)	0.00287(60)
*a*/*b*/*c* ^[c]^ [D]	m/w/s	m/–/s
*σ* ^[d]^ [kHz]	3.8	6.5
*N* ^[e]^	215	116

[a] *A*, *B* and *C* are the rotational constants. Δ_*J*_, Δ_*JK*_„ *δ_J_* are the quartic centrifugal distortion constants. [b] Standard error in parentheses in units of the last digit. [c] *a, b* and *c* are the type of transitions observed: strong, medium and weak. [d] *σ* is the r.m.s. deviation of the fit. [e] *N* is the number of fitted transitions.

**Table 4:** B3LYP‐D3BJ and MP2 spectroscopic parameters and relative energies for the isomers of the fenchone–benzene complex within 400 cm^−1^.

Isomer	**1**		**2**		**3**		**4**		**5**	
					
	B3LYP‐ D3BJ^[a]^	MP2	B3LYP‐ D3BJ	MP2	B3LYP‐ D3BJ	MP2	B3LYP‐ D3BJ	MP2	B3LYP‐ D3BJ	MP2
*A* ^[b]^ [MHz]	767.5	768.2	804.8	803.6	843.1	857.2	885.7	868.4	871.6	863.5
*B* [MHz]	342.1	354.4	323.2	332.5	308.5	316.4	281.8	294.7	295.7	308.4
*C* [MHz]	322.5	334.9	297.9	306.8	287.8	294.5	263.9	275.7	270.9	281.8
*μ_a_* [D]	1.0	0.8	−0.9	−0.8	−1.1	−1.1	−2.8	2.7	−2.9	−2.8
*μ_b_* [D]	−0.4	−0.4	0.1	−0.2	−1.0	−1.1	2.1	1.9	−1.9	1.8
*μ_c_* [D]	2.5	−2.3	2.6	2.4	2.3	−1.9	0.0	−0.2	0.4	0.2
Δ*E* ^[c]^ [cm^−1^]	0	0	180	384	324	539	338	523	341	433
Δ*E*_ZPE_^[d]^ [cm^−1^]	0	0	195	329	309	482	316	490	305	380
*D*_e_^[e]^ [kJ mol^−1^]	22.0	17.4	20.0	15.5	18.4	14.0	18.3	14.4	18.1	14.5
	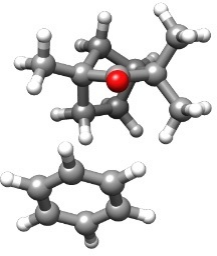		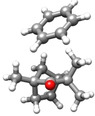		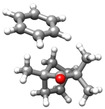		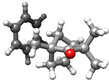		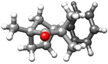	

[a] All calculations were performed using the 6‐311++G(d,p) basis set. [b] *A*, *B* and *C* are the rotational constants. *μ_a_*, *μ_b_* and *μ_c_* are the electric dipole moment components. [c] Relative electronic energies. [d] Relative electronic energies including the zero‐point correction. [e] Dissociation energies.

By measuring the intensities of several *c*‐type transitions and correcting them by the square of the predicted dipole moment components, the relative abundances of the observed isomers were estimated to be FBEN1/FBEN2=4:1 (see Figure S2 in the Supporting Information). This value is in good qualitative agreement with the relative energies and dissociation energies, including the basis set superposition error (BSSE) corrections, predicted by theoretical methods.

## Discussion

**Figure 1:**
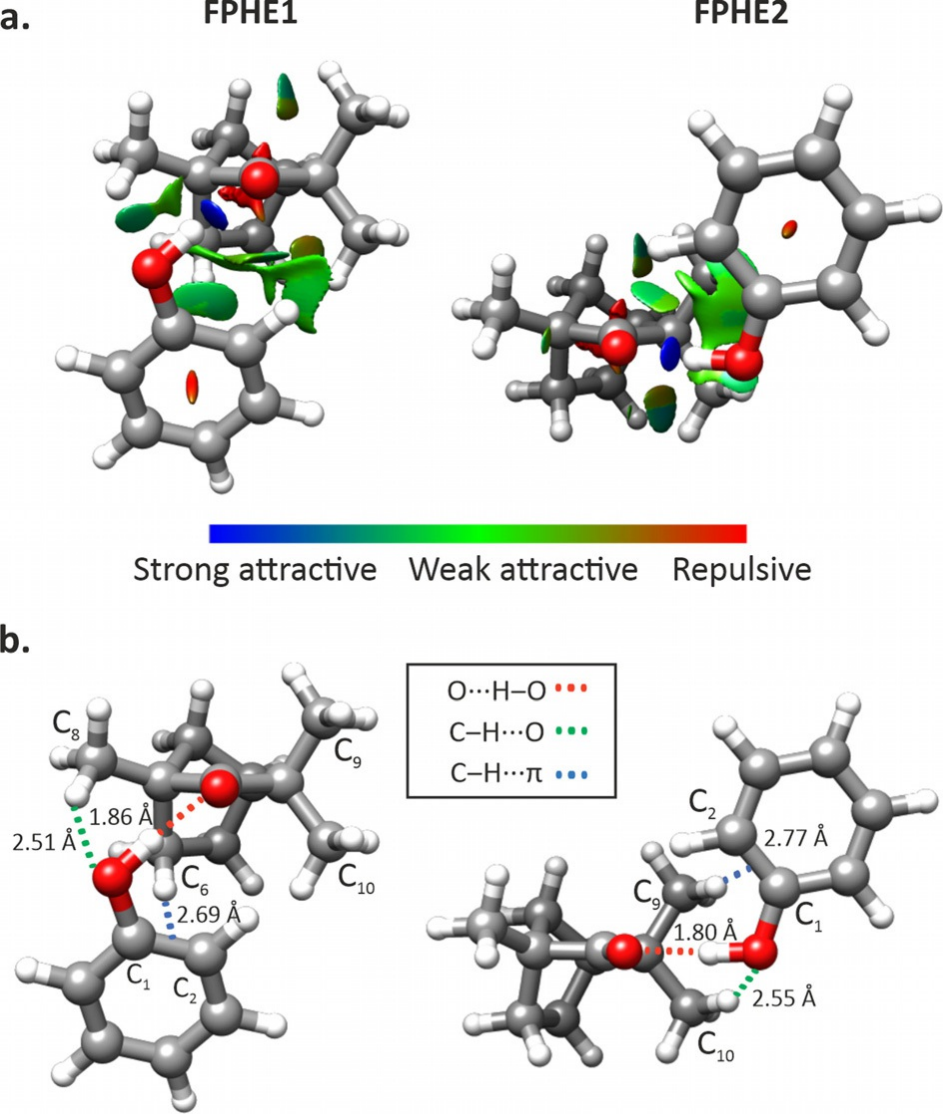
Observed isomers of fenchone–phenol. a) NCI plots and b) distances of the relevant intermolecular interactions determined at the B3LYP‐D3BJ/6‐311++G** level of theory.

**Figure 2:**
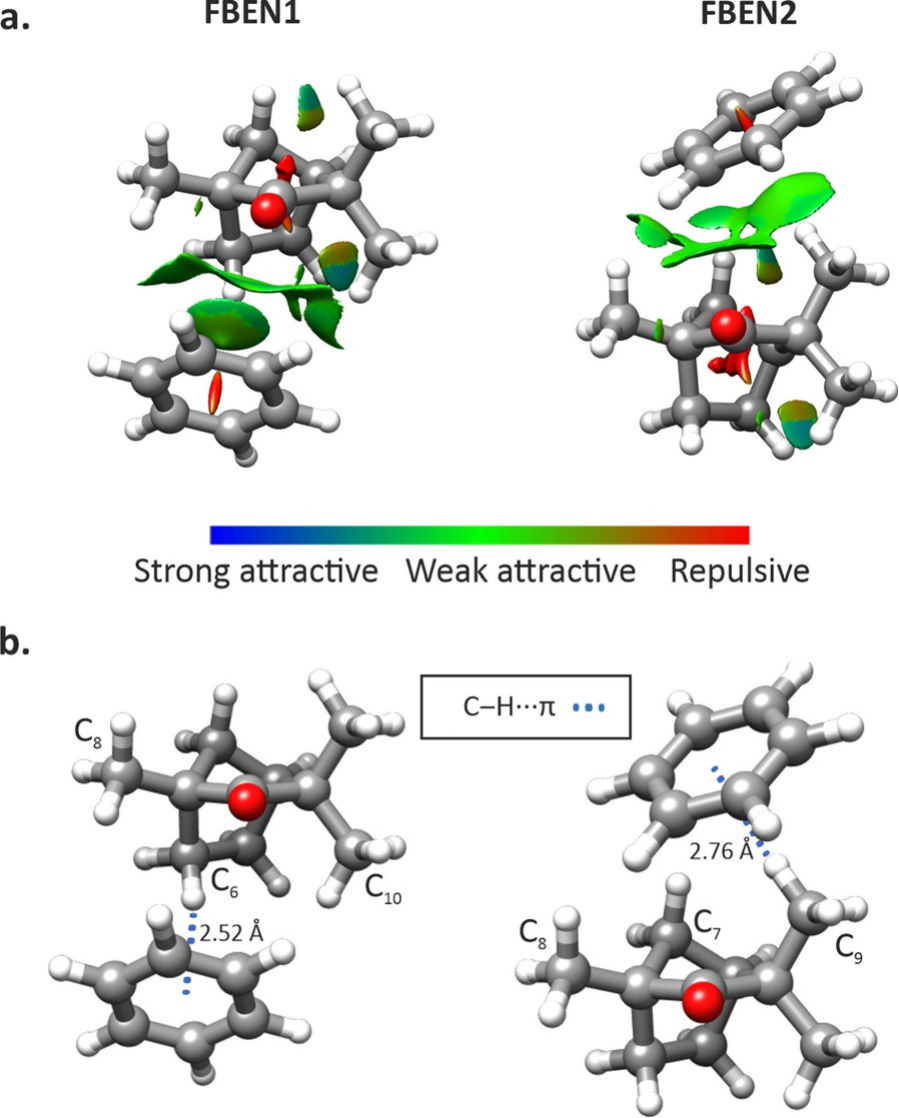
Observed isomers of fenchone–benzene. a) NCI plots and b) distances of the relevant intermolecular interactions determined at the B3LYP‐D3BJ/6–311++G** level of theory.

The interactions involved in stabilizing the observed isomers of fenchone–phenol and fenchone–benzene can be visualized by applying the non‐covalent interactions (NCIs) method^34^ to analyse the electron density and its derivatives (Figures 1 and 2). In fenchone—phenol, the dark‐blue disks indicate strong O−H⋅⋅⋅O hydrogen bonds between the oxygen of fenchone and the ‐OH group of phenol. The green isosurfaces indicate weak attractive interactions, that is, C−H⋅⋅⋅O hydrogen bonds involving the phenolic oxygen and the methyl groups of fenchone, and C−H⋅⋅⋅π bonds between one of the fenchone hydrogen atoms and the π electron density of the C1−C2 bond of phenol. Moreover, and perhaps surprisingly, there are also weak attractive interactions between the oxygen of fenchone and one of the hydrogen atoms of the aromatic ring in phenol. In addition, in FPHE1, there is a dispersion interaction between the hydrogen atoms of the methyl group of C10 and the closest hydrogen atoms of the aromatic ring in phenol. It is likely that this additional dispersive interaction, together with a more directed C−H⋅⋅⋅π bond, results in FPHE1 being the lowest‐energy isomer.

Fenchone–benzene complexes display very similar interactions, with green ‘umbrella’ isosurfaces indicating a primary C−H⋅⋅⋅π bond between the hydrogen atoms of the ‐CH_2_ and ‐CH_3_ groups in fenchone and the π electron density of benzene, and flat green isosurfaces indicating dispersive attractive interactions between the methyl hydrogen atoms of fenchone and the benzene ring (Figure 2). Similarly to fenchone–phenol, there are also weak interactions involving the oxygen atom of fenchone and the hydrogen atoms of benzene.

The different interactions involved in the stabilisation of fenchone–phenol and fenchone–benzene may explain the different results obtained by the theoretical methods for each complex. Although MP2, M06‐2X and B3LYP‐D3BJ generally agree in their predictions for fenchone–benzene, there are large discrepancies for fenchone–phenol, especially between B3LYP‐D3BJ and MP2. A comparison of the theoretical structures of fenchone–phenol (see Figure S3 in the Supporting Information) shows that the MP2 level predicts that the aromatic ring of phenol forms closer interactions with the C−H in fenchone and that the O−H⋅⋅⋅O and C−H⋅⋅⋅O bonds weaken. This changes the mass distribution along the principal inertial axes, which results in large differences in the MP2 rotational constants and dipole moment components with respect to those predicted by B3LYP‐D3BJ. In fenchone–benzene, the main interaction is a C−H⋅⋅⋅π bond, and there is no competition between intermolecular forces, which yields very similar structures by MP2 and B3LYP‐D3BJ (see Figure S4). The difficulties associated with balancing competing weak interactions by theoretical methods have been reported for other molecular systems, including the overestimation of C−H⋅⋅⋅π interactions by MP2 and challenges in describing the dispersion interactions.^35^, ^36^

Interestingly, although the primary interaction in the fenchone–phenol and fenchone–benzene complexes is different, the arrangement of phenol/benzene with respect to fenchone in their respective global minima, FPHE1 and FBEN1, is very similar, with their aromatic rings interacting with one of the C6 hydrogen atoms. This arrangement contrasts with the preferred binding site of the 1:1 complexes of fenchone with water^17^ and ethanol.^15^ In these two complexes, water and ethanol locate themselves on the other side of fenchone, with their ‐OH groups in the plane bisecting the C9‐C1‐C10 angle and binding through O−H⋅⋅⋅O hydrogen bonds to the oxygen in fenchone (see Figure S5 in the Supporting Information). This arrangement is similar to that in FPHE2 (Figure 1) and allows full overlap of the ‐OH group of phenol with the carbonyl oxygen lone pair. In FPHE1, the C8 methyl group of fenchone forces the O−H⋅⋅⋅O bond to be out‐of‐plane, thereby weakening the hydrogen bond and decreasing electrostatic contributions.

The different preference of binding site must be related to differences in the interactions established between the different moieties. How does the balance of forces change along the series water–ethanol–phenol–benzene? The energy decomposition analysis performed with symmetry‐adapted perturbation theory (SAPT)^37^, ^38^ shows that the contribution of electrostatic forces to the total attractive forces decreases from around 65 % in fenchone–water to around 30 % in fenchone–benzene, whereas dispersion interactions increase from approximately 17 to 60 % (Table 5), ultimately producing a change in the preferred binding site. The larger contribution of dispersion interactions in FPHE1 makes this isomer become the global minimum to the detriment of FPHE2.

**Table 5:** Binding energy decomposition in kJ mol^−1^ for the observed isomers of fenchone complexes on their B3LYP‐D3BJ/6‐311++G(d,p) geometries, using SAPT(0)/jun‐cc‐pDVZ calculations within Psi4.

	Δ*E*_electrostatic_	Δ*E*_exchange_	Δ*E*_induction_	Δ*E*_dispersion_	Δ*E*_total_
**fenchone–water^[a]^**					
1w‐I	−50.1 (65 %)^[b]^	43.6	−14.3 (18 %)	−12.9 (17 %)	−33.6
1w‐II	−43.5 (64 %)	37.5	−12.2 (18 %)	−12.3 (18 %)	−30.5

**fenchone–ethanol^[c]^**					
g+a1	−53.8 (60 %)	54.7	−16.9 (19 %)	−19.6 (22 %)	−35.5
g‐a2	−54.3 (60 %)	55.4	−17.1 (19 %)	−19.8 (22 %)	−35.8
g‐b	−47.9 (57 %)	51.0	−15.0 (18 %)	−21.7 (26 %)	−33.6

**fenchone–phenol^[d]^**					
FPHE1	−65.8 (52 %)	75.2	−21.2 (17 %)	−38.8 (31 %)	−50.6
FPHE2	−66.8 (57 %)	69.4	−23.4 (20 %)	−27.6 (23 %)	−48.4

**fenchone–benzene^[d]^**					
FBEN1	−18.7 (33 %)	34.9	−5.2 (9 %)	−33.2 (58 %)	−22.1
FBEN2	−15.3 (30 %)	32.4	−4.4 (9 %)	−31.5 (62 %)	−18.9

[a] Ref. 17. [b] Numbers in parentheses are the percentages of the total attractive interactions. [c] Ref. 15. [d] This work.

For the second‐lowest‐energy isomers, FPHE2 and FBEN2, the location of phenol/benzene is completely different. The position of phenol is determined by the formation of an O−H⋅⋅⋅O hydrogen bond, which constrains it to be close to the carbonyl group of fenchone. For fenchone–benzene, in which the main interaction is a C−H⋅⋅⋅π bond, there are fewer limitations, with the preferred benzene locations likely to be more dependent on secondary interactions.

The fact that fenchone–benzene is not bound by a strong O−H⋅⋅⋅O bond alters the energy balance with respect to the other fenchone complexes (Table 5). In fenchone–benzene, the electrostatic/dispersion ratio is almost reversed, with dispersion becoming the largest contributor to the attractive forces. The share of induction is significantly reduced to 9 % (from 19 % in other fenchone complexes), as expected in a complex in which one of the moieties does not have a dipole moment.

The strengths of the different interactions are revealed in the overall values of the binding energies (Table 5). They increase along the water–ethanol–phenol sequence, becoming significantly larger for fenchone–phenol, in line with the greater dispersion contributions. However, the binding energies for fenchone–benzene are the lowest. The larger dispersion values for fenchone–benzene cannot compensate the much decreased electrostatic and induction contributions.

Despite the significant chemical differences in the investigated partners binding to fenchone, all the complexes show a number of isomers very close in energy, within 4–5 kJ mol^−1^. The three‐dimensional structure of fenchone, with many hydrogen atoms available to establish secondary interactions, favours the existence of several contact points with interacting partners and therefore the appearance of multiple isomers with similar relative energies. This may have an impact on the ability of fenchone to interact with different odorant receptors.

Our results can be interpreted within the framework of intermolecular energy balances,^39^ in which carefully tuned substituents in the moieties forming the complex can tip the preferred interaction or binding site. In this context, different docking partners for fenchone can tune structural preferences, with preferences in binding site modulated by dispersion forces. It would be interesting to see whether substituted benzenes or other aromatics display the same docking preference observed for benzene, for which C−H⋅⋅⋅π and dispersion interactions drive binding. For complexes of ketones with water and alcohol, for which the same primary interaction O−H⋅⋅⋅O is observed, our work shows that changes in the alcohol modify the preference in the binding site due to increased dispersion interactions. This has also been observed for alcohol docking to substituted acetophenone, but in this case the two carbonyl binding sites are very different, as one involves a phenyl and the other an alkyl group.^40^ In other studies exploring the interactions of water and alcohols with ethers, changes in the preferred primary bonding, O−H⋅⋅⋅O versus O−H⋅⋅⋅π, were observed due to dispersion effects and structural flexibility.^41^, ^42^

The data presented here provide useful experimental benchmarks for computational methods, in particular for analysing and accurately describing the effects of dispersion and competing interactions. In this respect, B3LYP‐D3BJ is emerging as a reliable cost‐efficient method, whereas MP2 and M06‐2X exhibit deficiencies that impact on their predictions of the structures and energetics of molecular systems with several weak hydrogen bonds and dispersive interactions. Further experiments with a variety of spectroscopic methods are necessary to show the outcome of nuanced forces on prototype molecular systems, and to provide robust data to test the performance of theoretical methods and help develop new approaches.

## Conclusions

Two different isomers of fenchone–phenol and fenchone–benzene have been unambiguously identified by broadband rotational spectroscopy. The lowest‐energy isomers of the complexes show a very similar arrangement in which phenol and benzene prefer the same binding site of fenchone. This is in contrast to what was observed in the fenchone–water and fenchone–ethanol complexes. The increased contribution of dispersion interactions in the fenchone–phenol and fenchone–benzene complexes results in a switch in binding site preference. Our results illustrate the large differences that can arise from small changes in the balance of intermolecular forces and set the basis for a better understanding of the effect of different amino acid residues in molecular recognition and binding events.

## Experimental Section

**Theoretical**: The MMFF molecular mechanics method^43^ was used to explore the potential energy surfaces of the complexes, returning 34 and 31 structures for fenchone–phenol and fenchone–benzene, respectively, within 25 kJ mol^−1^. The resulting structures were subsequently optimized at the B3LYP‐D3BJ/6‐311++G(d,p) level of theory^44^, ^45^, ^46^ using tight optimization and an ultrafine grid, which yielded five distinct isomers for fenchone–phenol and 12 isomers for fenchone–benzene. Additional optimizations at the MP2^47^ and M06‐2X^48^ levels of theory with the 6‐311++G(d,p) basis set were carried out on the resulting structures. All isomers were confirmed to be local minima by performing harmonic vibrational calculations, and their zero‐point relative energies were obtained. Corrections to isomer energies due to BSSE were determined by using the counterpoise method^49^ including fragment relaxation terms^50^ and were used to calculate dissociation energies. All the results are shown in Tables 1 and 4 and Tables S1 and S7 in the Supporting Information. The B3LYP‐D3BJ and MP2 theoretical parameters for all five fenchone–phenol isomers and the five lowest‐energy isomers of fenchone–benzene are presented in Tables 1 and 4, respectively. For the fenchone–phenol complex, isomer 3 is a distinct isomer according to the B3LYP‐D3BJ and M06‐2X levels of theory, but converges to isomer 4 at the MP2 level.

**Experimental**: Commercial samples of (*R*)‐fenchone (Sigma–Aldrich, ≥98 %) and phenol (Sigma–Aldrich, unstabilised ≥99 %) were used to record the broadband microwave spectrum of the fenchone–phenol complex using our CP‐FTMW spectrometer operating in the 2–8 GHz frequency range.^16^, ^51^ Fenchone was placed in a bespoke heating nozzle inside the vacuum chamber at a temperature of around 358 K and phenol was placed in a heating reservoir in the injection line outside the chamber. Optimal signals were obtained when phenol was gently heated to 313 K. Both fenchone and phenol were seeded in neon at 5 bar and conducted to the vacuum chamber where they adiabatically expanded to form a supersonic jet. Complexes were produced by collisions at the beginning of the supersonic expansion, and they were subsequently polarized by four chirped microwave pulses of 4 μs duration each. After each microwave pulse, the molecular free induction decay (FID) was collected for 20 μs and transformed to the frequency domain using a fast Fourier transform algorithm. The spectrum of fenchone–benzene was recorded by using the same setup. Benzene (Sigma–Aldrich, anhydrous, 99.8 %) was placed in a separate reservoir in the injection line and held at room temperature. The final rotational spectra recorded for fenchone–phenol and fenchone–benzene have 2.3M and 3.6M FIDs, respectively.

## Conflict of interest

The authors declare no conflict of interest.

## Supporting information

As a service to our authors and readers, this journal provides supporting information supplied by the authors. Such materials are peer reviewed and may be re‐organized for online delivery, but are not copy‐edited or typeset. Technical support issues arising from supporting information (other than missing files) should be addressed to the authors.

Supplementary
