# Supplementary material for: Binding Site Switch by Dispersion Interactions: Rotational Signatures of Fenchone–Phenol and Fenchone–Benzene Complexes
Source: Chemistry. 2020 Aug 13;26(49):11327–33. doi: 10.1002/chem.202001713 (PMC7497235; doi:10.1002/chem.202001713)

Chemistry–A European Journal

Supporting Information

Binding Site Switch by Dispersion Interactions: Rotational Signatures of Fenchone–Phenol and Fenchone–Benzene Complexes

Ecaterina Burevschi, Elena R. Alonso, and M. Eugenia Sanz*[a]

Supplementary Information

Table S1. Predicted spectroscopic rotational parameters and relative energies for fenchone-phenol complexes at the M06-2X/6-311++G(d,p) level of theory.

Tables S2-S3. Measured frequencies and residuals of the rotational transitions of the fenchone-phenol complexes.

Table S4. Performance of the theoretical methods B3LYP-D3BJ, MP2 and M06-2X in percentage errors of the predicted rotational constants with respect to the experimental values.

Table S5-S6. Measured frequencies and residuals of the rotational transitions of the fenchone-benzene complexes.

Table S7. Predicted spectroscopic rotational parameters and relative energies for all fenchone-benzene complexes at B3LYP-D3BJ, M06-2X and MP2 level of theory with 6-311++G(d,p) basis set.

Tables S8-S11. B3LYP-D3BJ/6-311++G(d,p) structures of the observed isomers of fenchone-phenol and fenchone-benzene complexes.

Figure S1. Interconversion barriers between isomer 3 ↔ isomer 2 (top) and isomer 3 ↔ isomer 4 (bottom) of fenchone-phenol at the B3LYP-D3BJ level of theory.

Figure S2. Sections of the broadband rotational spectra of fenchone-phenol (left) and fenchone-benzene (right).

Figure S3-S4. Comparison between the B3LYP-GD3BJ and MP2 fenchone-phenol and fenchone-benzene structures (front and side views). All calculations used the 6-311++G(d,p) basis set.

Figure S5. Comparison between the lowest-energy isomers of the fenchone-water, fenchone-ethanol, fenchone-phenol and fenchone-benzene complexes.

Table S1. Predicted spectroscopic rotational parameters and relative energies for fenchone-phenol complexes at the M06-2X/6-311++G(d,p) level of theory.

	**1**	**2**	**3**	**4**	**5**
**A(MHz)**	683.7	909.6	849.6	697.9	767.5
**B(MHz)**	321.4	213.4	216.4	288.9	269.0
**C(MHz)**	310.5	199.7	211.3	272.1	257.7
**μ_a (D)**	3.0	-4.3	4.4	-3.5	3.6
**μ_b (D)**	1.9	0.2	-0.3	-1.7	0.8
**μ_c (D)**	2.3	-2.3	2.3	-2.3	-2.9
**ΔE(cm^{-1})**	0	371	361	319	441
**ΔE_{ZPE}(cm^{-1})**	0	413	332	363	536
**D_e (kJ/mol)**	45.2	42.1	42.3	41.9	40.7

Table S2. Measured frequencies and residuals (in MHz) of the rotational transitions of FPHE1.

J’	K’$_{-1}$	K’$_{+1}$	J’’	K’’$_{-1}$	K’’$_{+1}$	ν_{obs}	ν_{obs}- ν_{calc}
4	1	4	3	0	3	2583.1316	-0.0139
4	1	3	3	0	3	2734.9952	0.0056
5	1	5	4	1	4	2760.2811	0.0013
5	0	5	4	0	4	2791.8044	-0.0021
5	2	4	4	2	3	2799.0934	0.0093
5	4	2	4	4	1	2800.9553	-0.0023
5	4	1	4	4	0	2800.9553	-0.0031
5	3	3	4	3	2	2801.3724	0.0048
5	3	2	4	3	1	2801.5145	-0.0008
5	2	3	4	2	2	2807.2639	-0.0036
5	1	4	4	1	3	2836.0769	-0.0087
3	2	2	2	1	1	2911.4535	0.0100
3	2	1	2	1	1	2913.5098	-0.0003
3	2	2	2	1	2	2957.0095	-0.0001
6	1	6	5	1	5	3311.2513	-0.0023
5	1	4	4	0	4	3335.1755	-0.0008
6	0	6	5	0	5	3345.7866	0.0004
6	2	5	5	2	4	3358.1024	0.0053
6	4	3	5	4	2	3361.4116	0.0063
6	4	2	5	4	1	3361.4116	0.0027
6	3	4	5	3	3	3362.0623	0.0040
6	3	3	5	3	2	3362.4511	-0.0005
6	2	4	5	2	3	3372.2516	0.0025
6	1	5	5	1	4	3402.0129	0.0022
4	2	3	3	1	2	3448.5943	-0.0034
4	2	2	3	1	2	3454.7858	0.0026
4	2	3	3	1	3	3539.7246	0.0002
7	0	7	6	1	6	3616.4490	0.0000
6	1	6	5	0	5	3626.9502	-0.0234
3	3	1	2	2	1	3770.5962	-0.0094
7	1	7	6	1	6	3861.6984	0.0016
7	0	7	6	0	6	3897.6412	0.0050
7	2	6	6	2	5	3916.6517	-0.0213
7	5	3	6	5	2	3921.4408	-0.0004
7	5	2	6	5	1	3921.4408	-0.0005
7	4	4	6	4	3	3922.0011	0.0099
7	4	3	6	4	2	3922.0011	-0.0020
7	3	5	6	3	4	3922.9195	0.0028
7	3	4	6	3	3	3923.7985	-0.0006
7	2	5	6	2	4	3938.8940	0.0017
6	1	5	5	0	5	3945.3771	-0.0034
7	1	6	6	1	5	3967.1660	0.0012
5	2	4	4	1	3	3978.1333	0.0030
5	2	3	4	1	3	3992.4955	-0.0037
5	2	4	4	1	4	4129.9712	-0.0031
7	1	7	6	0	6	4142.8875	0.0033

8	0	8	7	1	7	4202.0606	-0.0011
4	3	2	3	2	2	4331.4278	0.0116
8	1	8	7	1	7	4411.5701	0.0027
8	0	8	7	0	7	4447.3066	-0.0029
8	2	7	7	2	6	4474.7272	-0.0142
8	6	3	7	6	2	4481.5031	0.0067
8	6	2	7	6	1	4481.5031	0.0067
8	5	4	7	5	3	4481.9126	0.0043
8	5	3	7	5	2	4481.9126	0.0040
8	4	5	7	4	4	4482.7497	0.0140
8	4	4	7	4	3	4482.7497	-0.0187
8	3	6	7	3	5	4483.9285	0.0043
8	3	5	7	3	4	4485.6830	0.0020
8	2	6	7	2	5	4507.1585	-0.0040
6	2	4	5	1	4	4528.6614	-0.0013
8	1	7	7	1	6	4531.3566	-0.0082
7	1	6	6	0	6	4566.7506	-0.0085
8	1	8	7	0	7	4656.8164	0.0011
6	2	5	5	1	5	4727.7874	-0.0042
9	0	9	8	1	8	4785.4369	0.0052
5	3	3	4	2	3	4893.0752	-0.0024
9	1	9	8	1	8	4960.8425	0.0011
9	0	9	8	0	8	4994.9352	-0.0022
9	2	8	8	2	7	5032.2342	0.0001
9	6	4	8	6	3	5041.8838	0.0056
9	6	3	8	6	2	5041.8838	0.0056
9	5	5	8	5	4	5042.4818	0.0017
9	5	4	8	5	3	5042.4818	0.0008
9	4	6	8	4	5	5043.6935	0.0365
9	4	5	8	4	4	5043.6935	-0.0419
9	3	7	8	3	6	5045.0481	0.0040
9	3	6	8	3	5	5048.2438	0.0004
7	2	5	6	1	5	5065.5423	-0.0020
9	2	7	8	2	6	5076.8447	0.0013
9	1	8	8	1	7	5094.4025	-0.0016
8	1	7	7	0	7	5200.4899	0.0022
7	2	6	6	1	6	5333.2144	0.0035
10	0	10	9	1	9	5365.4072	0.0016
6	3	4	5	2	4	5456.0497	-0.0020
10	1	10	9	1	9	5509.5159	0.0038
8	2	7	7	1	6	5522.3844	0.0039
10	0	10	9	0	9	5540.8123	-0.0030
10	2	9	9	2	8	5589.0871	0.0010
10	6	5	9	6	4	5602.3100	-0.0190
10	6	4	9	6	3	5602.3100	-0.0191
10	5	6	9	5	5	5603.1715	0.0018
10	5	5	9	5	4	5603.1715	-0.0008
10	4	7	9	4	6	5604.7778	0.0077

10	4	6	9	4	5	5604.9339	-0.0058
8	2	6	7	1	6	5605.5454	0.0035
10	3	8	9	3	7	5606.2251	0.0029
10	3	7	9	3	6	5611.6541	-0.0006
10	2	8	9	2	7	5647.5517	0.0017
10	1	9	9	1	8	5656.0549	0.0015
10	1	10	9	0	9	5684.9272	0.0053
9	1	8	8	0	8	5847.5858	0.0035
8	2	7	7	1	7	5946.2534	-0.0021
7	3	4	6	2	4	5993.8249	0.0019
7	3	5	6	2	5	6020.8709	-0.0004
9	2	8	8	1	7	6023.2446	-0.0052
11	1	11	10	1	10	6057.5836	-0.0054
11	0	11	10	0	10	6085.3504	0.0056
11	2	10	10	2	9	6145.2371	0.0002
9	2	7	8	1	7	6151.0224	0.0019
11	6	6	10	6	5	6162.8596	0.0030
11	6	5	10	6	4	6162.8596	0.0029
11	5	7	10	5	6	6163.9956	0.0053
11	5	6	10	5	5	6163.9956	-0.0010
11	4	8	10	4	7	6166.0864	0.0011
11	4	7	10	4	6	6166.4222	-0.0009
11	3	9	10	3	8	6167.3862	-0.0022
11	3	8	10	3	7	6176.0917	-0.0085
11	1	11	10	0	10	6201.6946	-0.0010
11	1	10	10	1	9	6216.0693	0.0032
11	2	9	10	2	8	6218.7839	0.0002
10	1	9	9	0	9	6508.6957	-0.0026
10	2	9	9	1	8	6517.9364	0.0046
8	3	5	7	2	5	6540.6104	-0.0014
9	2	8	8	1	8	6566.9281	0.0059
8	3	6	7	2	6	6588.1235	0.0009
12	1	12	11	1	11	6605.0974	0.0020
12	0	12	11	0	11	6628.9511	-0.0043
12	2	11	11	2	10	6700.6353	0.0033
10	2	8	9	1	8	6704.1681	0.0017
12	6	7	11	6	6	6723.4839	0.0149
12	6	6	11	6	5	6723.4839	0.0148
12	5	8	11	5	7	6724.9561	0.0014
12	5	7	11	5	6	6724.9561	-0.0130
12	4	9	11	4	8	6727.6081	0.0014
12	4	8	11	4	7	6728.2406	0.0037
12	3	10	11	3	9	6728.4554	-0.0031
12	3	9	11	3	8	6741.7723	0.0015
12	1	11	11	1	10	6774.1896	0.0006
12	2	10	11	2	9	6790.0102	0.0006
9	3	6	8	2	6	7081.6895	-0.0032
13	1	13	12	1	12	7152.0762	0.0104

13	0	13	12	0	12	7172.0355	0.0041
10	2	9	9	1	9	7195.1672	0.0003
13	2	12	12	2	11	7255.2328	0.0081
11	2	9	10	1	9	7266.9043	0.0077
13	6	8	12	6	7	7284.1706	-0.0034
13	6	7	12	6	6	7284.1706	-0.0039
13	5	9	12	5	8	7286.0923	0.0170
13	5	8	12	5	7	7286.0923	-0.0137
13	3	11	12	3	10	7289.3385	0.0005
13	4	10	12	4	9	7289.3385	0.0080
13	4	9	12	4	8	7290.4455	0.0016
13	3	10	12	3	9	7308.8395	-0.0023
13	1	12	12	1	11	7330.1849	0.0017
13	2	11	12	2	10	7360.7251	-0.0006
14	1	14	13	1	13	7698.5356	-0.0059
14	0	14	13	0	13	7714.8554	-0.0134
14	2	13	13	2	12	7808.9870	0.0099
11	2	10	10	1	10	7830.8874	-0.0044
12	2	10	11	1	10	7840.8427	0.0026
14	6	9	13	6	8	7844.9695	-0.0105
14	6	8	13	6	7	7844.9695	-0.0116
14	3	12	13	3	11	7849.9199	-0.0053
14	4	11	13	4	10	7851.2461	0.0025
14	4	10	13	4	9	7853.1147	-0.0063
14	3	11	13	3	10	7877.4391	-0.0038
14	1	13	13	1	12	7883.8549	0.0013
14	2	12	13	2	11	7930.5035	0.0000

Table S3. Measured frequencies and residuals (in MHz) of the rotational transitions of FPHE2.

J'	K'_{-1}	K'_{+1}	J''	K''_{-1}	K''_{+1}	ν_{obs}	ν_{obs}- ν_{calc}
7	1	7	6	1	6	2745.4631	-0.0047
7	0	7	6	0	6	2783.5514	-0.0021
7	2	6	6	2	5	2792.1714	0.0009
7	4	4	6	4	3	2794.4931	0.0050
7	4	3	6	4	2	2794.4931	0.0036
7	2	5	6	2	4	2802.1841	-0.0052
5	1	4	4	0	4	2805.7438	0.0026
7	1	6	6	1	5	2836.9381	0.0087
2	2	1	1	1	1	2936.7721	-0.0090
8	1	8	7	1	7	3136.8410	-0.0001
8	0	8	7	0	7	3177.7279	0.0019
8	2	7	7	2	6	3190.4373	0.0016
8	5	4	7	5	3	3193.5474	-0.0045
8	5	3	7	5	2	3193.5474	-0.0045
8	4	5	7	4	4	3193.9389	0.0119
8	4	4	7	4	3	3193.9389	0.0082
8	3	6	7	3	5	3194.6275	-0.0011
8	3	5	7	3	4	3195.0220	0.0008
8	2	6	7	2	5	3205.3514	0.0038
8	1	7	7	1	6	3241.1978	-0.0028
6	1	5	5	0	5	3246.2198	0.0022
3	2	1	2	1	1	3310.5859	0.0017
3	2	2	2	1	2	3348.9692	0.0073
9	1	9	8	1	8	3527.9078	-0.0020
9	0	9	8	0	8	3570.6277	0.0007
9	2	8	8	2	7	3588.4555	0.0002
9	5	5	8	5	4	3592.9180	-0.0022
9	5	4	8	5	3	3592.9180	-0.0023
9	4	6	8	4	5	3593.4641	0.0086
9	4	5	8	4	4	3593.4641	-0.0003
9	3	7	8	3	6	3594.3819	0.0010
9	3	6	8	3	5	3595.1008	0.0012
9	2	7	8	2	6	3609.5234	0.0001
9	1	8	8	1	7	3645.0398	0.0025
4	2	2	3	1	2	3691.8137	0.0052
7	1	6	6	0	6	3694.9458	0.0051
4	2	3	3	1	3	3767.6630	0.0035
10	1	10	9	1	9	3918.6573	0.0037
10	0	10	9	0	9	3962.2031	-0.0012
10	2	9	9	2	8	3986.2001	0.0008
10	6	5	9	6	4	3991.9636	-0.0057
10	6	4	9	6	3	3991.9636	-0.0057
10	5	6	9	5	5	3992.3521	0.0020
10	5	5	9	5	4	3992.3521	0.0018
10	4	7	9	4	6	3993.0953	0.0112
10	4	6	9	4	5	3993.0953	-0.0080

10	3	8	9	3	7	3994.2411	-0.0009
10	3	7	9	3	6	3995.4725	0.0015
10	2	8	9	2	7	4014.7345	-0.0011
10	1	9	9	1	8	4048.3651	0.0004
5	2	3	4	1	3	4068.2757	0.0002
8	1	7	7	0	7	4152.5929	0.0050
5	2	4	4	1	4	4192.8906	0.0053
11	1	11	10	1	10	4309.0575	-0.0002
11	0	11	10	0	10	4352.4607	0.0015
11	2	10	10	2	9	4383.6403	0.0019
11	6	6	10	6	5	4391.3366	-0.0017
11	6	5	10	6	4	4391.3366	-0.0017
11	5	7	10	5	6	4391.8498	0.0013
11	5	6	10	5	5	4391.8498	0.0009
11	4	8	10	4	7	4392.8419	0.0194
11	4	7	10	4	6	4392.8419	-0.0189
11	3	9	10	3	8	4394.2022	-0.0002
11	3	8	10	3	7	4396.1927	-0.0003
11	2	9	10	2	8	4420.9297	-0.0199
6	2	4	5	1	4	4440.9053	-0.0028
11	1	10	10	1	9	4451.1018	0.0007
9	1	8	8	0	8	4619.8961	-0.0031
6	2	5	5	1	5	4624.6669	0.0058
12	1	12	11	1	11	4699.1111	-0.0018
12	0	12	11	0	11	4741.4518	0.0019
12	2	11	11	2	10	4780.7473	0.0032
12	6	7	11	6	6	4790.7589	0.0025
12	6	6	11	6	5	4790.7589	0.0025
12	5	8	11	5	7	4791.4272	0.0051
12	5	7	11	5	6	4791.4272	0.0042
12	4	9	11	4	8	4792.6949	0.0151
12	4	8	11	4	7	4792.6949	-0.0567
12	3	10	11	3	9	4794.2491	0.0018
12	3	9	11	3	8	4797.3254	-0.0050
7	2	5	6	1	5	4810.8054	0.0001
12	2	10	11	2	9	4828.0689	-0.0034
12	1	11	11	1	10	4853.1564	-0.0010
7	2	6	6	1	6	5063.0213	0.0050
13	1	13	12	1	12	5088.8152	-0.0002
10	1	9	9	0	9	5097.6390	0.0020
13	0	13	12	0	12	5129.2896	0.0012
13	2	12	12	2	11	5177.4923	0.0036
8	2	6	7	1	6	5179.2235	0.0000
13	6	8	12	6	7	5190.2381	0.0098
13	6	7	12	6	6	5190.2381	0.0098
13	5	9	12	5	8	5191.0828	0.0049
13	5	8	12	5	7	5191.0828	0.0031
13	4	10	12	4	9	5192.6640	-0.0001

13	4	9	12	4	8	5192.8016	0.0102
13	3	11	12	3	10	5194.3561	-0.0005
13	3	10	12	3	9	5198.9548	-0.0004
13	2	11	12	2	10	5235.9518	-0.0038
13	1	12	12	1	11	5254.4337	-0.0038
14	1	14	13	1	13	5478.1705	0.0038
8	2	7	7	1	7	5507.9876	0.0034
14	0	14	13	0	13	5516.1309	0.0006
5	3	2	4	2	2	5547.3980	-0.0052
9	2	7	8	1	7	5547.5411	-0.0050
5	3	3	4	2	3	5550.0762	0.0052
14	2	13	13	2	12	5573.8486	0.0026
11	1	10	10	0	10	5586.5344	0.0006
14	6	9	13	6	8	5589.7730	0.0147
14	6	8	13	6	7	5589.7730	0.0147
14	5	10	13	5	9	5590.8207	-0.0020
14	5	9	13	5	8	5590.8207	-0.0055
14	4	11	13	4	10	5592.7834	0.0013
14	3	12	13	3	11	5594.5073	0.0027
14	3	11	13	3	10	5601.1492	0.0036
14	2	12	13	2	11	5644.4076	0.0016
14	1	13	13	1	12	5654.8290	-0.0097
15	1	15	14	1	14	5867.1738	0.0006
15	0	15	14	0	14	5902.1475	-0.0108
10	2	8	9	1	8	5917.2419	-0.0024
6	3	3	5	2	3	5944.3164	-0.0015
6	3	4	5	2	4	5950.5034	-0.0029
9	2	8	8	1	8	5959.6021	0.0037
15	2	14	14	2	13	5969.7822	-0.0089
15	6	10	14	6	9	5989.3598	0.0087
15	6	9	14	6	8	5989.3598	0.0086
15	5	11	14	5	10	5990.6669	0.0037
15	5	10	14	5	9	5990.6669	-0.0029
15	4	12	14	4	11	5993.0288	-0.0103
15	4	11	14	4	10	5993.4026	0.0111
15	3	13	14	3	12	5994.6661	0.0051
15	3	12	14	3	11	6003.9745	-0.0098
15	2	13	14	2	12	6053.2026	0.0023
15	1	14	14	1	13	6054.2411	-0.0127
12	1	11	11	0	11	6087.2261	-0.0058
16	1	16	15	1	15	6255.8477	0.0021
11	2	9	10	1	9	6289.8219	-0.0072
7	3	4	6	2	4	6339.5186	-0.0005
7	3	5	6	2	5	6351.8023	-0.0027
16	2	15	15	2	14	6365.3019	0.0007
16	3	13	15	3	12	6407.5472	-0.0089
10	2	9	9	1	9	6417.8955	0.0076
16	1	15	15	1	14	6452.5866	0.0136

16	2	14	15	2	13	6462.1034	-0.0005
4	4	0	3	3	0	6571.1482	0.0014
4	4	1	3	3	1	6571.1482	-0.0002
13	1	12	12	0	12	6600.2222	0.0027
17	1	17	16	1	16	6644.1910	-0.0073
12	2	10	11	1	10	6666.7947	-0.0056
17	0	17	16	0	16	6672.5530	0.0220
8	3	5	7	2	5	6732.3569	0.0059
8	3	6	7	2	6	6754.2593	-0.0038
17	2	16	16	2	15	6760.3522	-0.0035
17	6	12	16	6	11	6788.7279	-0.0152
17	5	13	16	5	12	6790.6737	0.0155
17	6	13	16	6	12	6790.6737	-0.0057
17	4	14	16	4	13	6793.9662	-0.0144
17	3	15	16	3	14	6794.8568	0.0001
17	3	14	16	3	13	6811.9553	0.0120
17	1	16	16	1	15	6849.6757	-0.0134
5	4	1	4	3	1	6970.2797	0.0111
5	4	2	4	3	2	6970.2797	0.0003
18	1	18	17	1	17	7032.2708	0.0221
13	2	11	12	1	11	7049.6061	0.0075
18	0	18	17	0	17	7057.2242	0.0041
9	3	6	8	2	6	7122.1025	-0.0006
9	3	7	8	2	7	7158.2010	-0.0073
18	1	17	17	1	16	7245.4960	-0.0061
18	2	16	17	2	15	7279.3399	0.0014
6	4	2	5	3	2	7369.3493	0.0202
6	4	3	5	3	3	7369.3493	-0.0231
14	2	12	13	1	12	7439.5497	-0.0174
10	3	8	9	2	8	7563.9935	-0.0015

Table S4. Performance of the theoretical methods B3LYP-D3BJ, MP2 and M06-2X in percentage errors of the predicted equilibrium rotational constants with respect to the experimental values.

	FPHE1			FPHE2		
	**B3LYP-D3BJ**	**MP2**	**M06-2X**	**B3LYP-D3BJ**	**MP2**	**M06-2X**
**ΔA(MHz)**	0.1%[a]	-2.9%	-2.1%	-0.7%	-6.2%	-0.1%
**ΔB(MHz)**	2.5%	13.0%	11.8%	3.0%	11.1%	3.5%
**ΔC(MHz)**	2.5%	15.4%	14.0%	3.8%	11.6%	3.5%
	**FBEN1**			**FBEN2**		
	**B3LYP-D3BJ**	**MP2**	**M06-2X**	**B3LYP-D3BJ**	**MP2**	**M06-2X**
**ΔA(MHz)**	0.0%	0.1%	0.7%	-0.1%	-0.2%	0.8%
**ΔB(MHz)**	2.1%	5.8%	5.4%	2.7%	5.7%	6.2%
**ΔC(MHz)**	2.0%	5.9%	5.3%	2.7%	5.8%	5.9%

[a] Calculated as $(A_{calc}-A_{exp})/A_{exp}\times100\%$.

Table S5. Measured frequencies and residuals (in MHz) of the rotational transitions of FBEN1.

J'	K'_{-1}	K'_{+1}	J''	K''_{-1}	K''_{+1}	ν_{obs}	ν_{obs}- ν_{calc}
5	1	4	4	2	2	2059.3456	-0.0040
4	0	4	3	1	2	2097.5395	-0.0016
7	2	6	6	3	4	2333.3895	0.0080
6	1	6	5	2	4	2376.2502	0.0023
7	2	5	6	3	3	2406.0767	-0.0050
3	1	2	2	0	2	2452.4471	0.0014
4	1	4	3	1	3	2565.8093	-0.0003
4	0	4	3	0	3	2598.7896	0.0002
4	2	3	3	2	2	2604.3526	0.0008
4	3	1	3	3	0	2606.0703	-0.0050
4	2	2	3	2	1	2610.3884	0.0012
2	2	0	1	1	0	2619.1065	0.0011
2	2	1	1	1	1	2637.4149	-0.0016
2	2	0	1	1	1	2638.0292	0.0053
4	1	3	3	1	2	2641.4088	-0.0005
5	0	5	4	1	3	2700.1942	-0.0002
6	1	5	5	2	3	2751.4839	0.0029
4	1	4	3	0	3	2953.5636	0.0036
7	1	7	6	2	5	2956.7218	-0.0011
8	2	7	7	3	5	2972.8624	0.0042
11	4	8	11	3	8	3047.7735	0.0001
10	4	7	10	3	7	3063.6887	-0.0051
9	4	6	9	3	6	3074.5885	-0.0124
8	4	5	8	3	5	3081.8381	0.0080
8	2	6	7	3	4	3090.4515	0.0051
4	1	3	3	0	3	3142.6575	-0.0001
5	1	5	4	1	4	3206.1962	0.0001
5	0	5	4	0	4	3244.0670	0.0044
3	2	1	2	1	1	3253.8173	0.0000
5	2	4	4	2	3	3254.6733	0.0003
5	4	1	4	4	0	3257.3704	-0.0004
5	4	2	4	4	1	3257.3704	0.0012
5	3	2	4	3	1	3258.2349	-0.0042
5	2	3	4	2	2	3266.6332	0.0011
6	0	6	5	1	4	3286.2848	0.0008
5	1	4	4	1	3	3300.5308	-0.0002
3	2	2	2	1	2	3307.5415	0.0013
3	2	1	2	1	2	3310.5709	-0.0016
7	1	6	6	2	4	3442.2726	0.0043
8	1	8	7	2	6	3526.2593	-0.0029
5	1	5	4	0	4	3560.9656	-0.0012
9	2	8	8	3	6	3607.3429	0.0048
9	2	7	8	3	5	3785.0191	-0.0034
5	1	4	4	0	4	3844.4000	0.0009
6	1	6	5	1	5	3845.9354	-0.0005
7	0	7	6	1	5	3853.7958	0.0014

4	2	2	3	1	2	3882.5905	-0.0002
6	0	6	5	0	5	3886.6213	0.0008
6	2	5	5	2	4	3904.4854	0.0008
6	5	2	5	5	1	3908.7288	0.0001
6	5	1	5	5	0	3908.7288	0.0000
6	4	2	5	4	1	3909.2776	-0.0043
6	4	3	5	4	2	3909.2776	0.0030
6	3	4	5	3	3	3910.2244	0.0027
6	3	3	5	3	2	3910.8988	-0.0025
6	2	4	5	2	3	3925.0711	-0.0021
6	1	5	5	1	4	3958.7655	0.0019
4	2	3	3	1	3	3987.0206	-0.0002
4	2	2	3	1	3	3996.0963	0.0078
9	1	9	8	2	7	4084.8026	0.0011
8	1	7	7	2	5	4128.0201	0.0037
3	3	0	2	2	0	4162.6312	-0.0030
6	1	6	5	0	5	4162.8403	0.0003
3	3	1	2	2	1	4163.2266	-0.0027
10	2	9	9	3	7	4235.7914	-0.0024
8	0	8	7	1	6	4401.0396	0.0007
7	1	7	6	1	6	4484.9610	0.0014
10	2	8	9	3	6	4489.6209	0.0023
5	2	3	4	1	3	4507.8138	0.0003
7	0	7	6	0	6	4526.2730	-0.0009
7	2	6	6	2	5	4553.6891	0.0020
6	1	5	5	0	5	4559.1016	0.0016
7	5	3	6	5	2	4560.5521	0.0006
7	5	2	6	5	1	4560.5521	0.0004
7	4	3	6	4	2	4561.4248	-0.0114
7	4	4	6	4	3	4561.4248	0.0129
7	3	5	6	3	4	4562.7084	0.0007
7	3	4	6	3	3	4564.2311	0.0007
7	2	5	6	2	4	4585.7918	-0.0003
7	1	6	6	1	5	4615.8617	0.0010
10	1	10	9	2	8	4632.3792	-0.0028
5	2	4	4	1	4	4675.8842	-0.0001
7	1	7	6	0	6	4761.1750	-0.0042
9	1	8	8	2	6	4804.9056	0.0012
4	3	1	3	2	1	4812.6736	0.0110
4	3	2	3	2	2	4815.5982	-0.0114
12	6	7	12	5	7	4851.3619	0.0408
12	6	6	12	5	8	4851.3619	-0.0189
11	6	6	11	5	6	4853.6660	0.0170
11	6	5	11	5	7	4853.6660	-0.0076
10	6	5	10	5	5	4855.4416	0.0079
10	6	4	10	5	6	4855.4416	-0.0014
9	6	3	9	5	5	4856.7777	0.0036
9	6	4	9	5	4	4856.7779	0.0069

8	6	3	8	5	3	4857.7415	-0.0030
8	6	2	8	5	4	4857.7415	-0.0039
7	6	2	7	5	2	4858.4262	-0.0021
7	6	1	7	5	3	4858.4262	-0.0023
6	6	1	6	5	1	4858.8883	0.0016
6	6	0	6	5	2	4858.8883	0.0015
9	0	9	8	1	7	4926.9724	0.0012
8	1	8	7	1	7	5123.2283	0.0019
6	2	4	5	1	4	5132.3551	-0.0007
8	0	8	7	0	7	5163.1092	0.0040
11	1	11	10	2	9	5169.1654	-0.0018
8	2	7	7	2	6	5202.1843	0.0000
8	5	4	7	5	3	5212.5436	-0.0006
8	5	3	7	5	2	5212.5436	-0.0013
8	3	6	7	3	5	5215.4016	-0.0030
8	3	5	7	3	4	5218.4303	0.0004
8	2	6	7	2	5	5248.5964	0.0013
8	1	7	7	1	6	5271.5372	-0.0029
7	1	6	6	0	6	5288.3445	0.0043
6	2	5	5	1	5	5374.1733	0.0007
6	2	4	5	1	5	5415.7726	-0.0155
10	0	10	9	1	8	5431.4285	0.0032
5	3	2	4	2	2	5460.5189	0.0044
5	3	3	4	2	3	5469.2401	-0.0013
10	1	9	9	2	7	5469.2401	0.0016
12	1	12	11	2	10	5695.4527	0.0015
4	4	1	3	3	1	5697.8433	-0.0049
4	4	0	3	3	1	5697.8433	-0.0051
4	4	0	3	3	0	5697.8433	0.0071
4	4	1	3	3	0	5697.8433	0.0073
11	7	5	11	6	5	5739.5728	0.0028
11	7	4	11	6	6	5739.5728	0.0025
9	7	3	9	6	3	5741.4209	0.0100
9	7	2	9	6	4	5741.4209	0.0099
8	7	2	8	6	2	5741.9883	0.0007
8	7	1	8	6	3	5741.9883	0.0007
7	7	1	7	6	1	5742.3938	0.0003
7	7	0	7	6	2	5742.3938	0.0003
7	2	5	6	1	5	5759.3822	-0.0021
9	1	9	8	1	8	5760.7239	0.0003
9	0	9	8	0	8	5797.4692	-0.0032
9	2	8	8	2	7	5849.8868	0.0023
9	5	5	8	5	4	5864.7341	0.0029
9	5	4	8	5	3	5864.7341	0.0008
9	3	7	8	3	6	5868.2455	-0.0015
9	3	6	8	3	5	5873.7363	-0.0037
9	2	7	8	2	6	5913.0056	-0.0005
11	0	11	10	1	9	5915.2187	-0.0007

9	1	8	8	1	7	5925.4840	0.0009
9	1	9	8	0	8	5955.7448	-0.0054
8	1	7	7	0	7	6033.6068	0.0006
7	2	6	6	1	6	6081.9232	-0.0006
6	3	3	5	2	3	6104.7844	0.0008
11	1	10	10	2	8	6117.6593	0.0094
6	3	4	5	2	4	6124.7869	-0.0032
7	2	5	6	1	6	6155.6440	-0.0002
5	4	2	4	3	2	6349.1811	-0.0341
5	4	1	4	3	2	6349.1811	-0.0359
5	4	1	4	3	1	6349.1811	0.0494
5	4	2	4	3	1	6349.1811	0.0512
8	2	6	7	1	6	6392.1169	-0.0019
10	1	10	9	1	9	6397.4649	-0.0002
10	0	10	9	0	9	6429.9363	-0.0008
10	2	9	9	2	8	6496.7015	-0.0012
10	5	6	9	5	5	6517.1375	0.0010
10	5	5	9	5	4	6517.1375	-0.0051
10	4	7	9	4	6	6519.5255	0.0022
10	4	6	9	4	5	6519.8609	-0.0069
10	3	8	9	3	7	6521.1405	-0.0024
10	3	7	9	3	6	6530.4299	-0.0006
10	1	10	9	0	9	6555.7420	-0.0009
10	1	9	9	1	8	6577.3358	-0.0044
10	2	8	9	2	7	6578.3322	-0.0039
12	8	5	12	7	5	6623.4446	-0.0124
11	8	4	11	7	4	6624.3384	0.0047
10	8	3	10	7	3	6625.0048	-0.0051
9	8	2	9	7	2	6625.5173	-0.0017
8	8	1	8	7	1	6625.8879	-0.0033
7	3	4	6	2	4	6743.9420	0.0013
12	1	11	11	2	9	6747.1991	0.0001
7	3	5	6	2	5	6783.0112	-0.0021
9	1	8	8	0	8	6795.9830	-0.0013
8	2	7	7	1	7	6799.1486	0.0001
9	2	7	8	1	7	7033.5770	-0.0078
11	0	11	10	0	10	7061.1280	-0.0063
11	2	10	10	2	9	7142.5644	0.0016
11	5	7	10	5	6	7169.7892	0.0050
11	5	6	10	5	5	7169.7892	-0.0102
11	4	8	10	4	7	7172.8645	-0.0003
11	4	7	10	4	6	7173.5523	0.0024
11	3	9	10	3	8	7173.9775	-0.0002
11	3	8	10	3	7	7188.7817	-0.0034
11	1	10	10	1	9	7226.7462	-0.0012
5	5	0	4	4	0	7232.7336	-0.0003
5	5	1	4	4	0	7232.7336	-0.0003
5	5	0	4	4	1	7232.7336	-0.0005

5	5	1	4	4	1	7232.7336	-0.0005
11	2	9	10	2	8	7243.8132	0.0041
8	3	5	7	2	5	7376.5824	0.0038
8	3	6	7	2	6	7444.7300	-0.0008
9	2	8	8	1	8	7525.8115	0.0049
10	1	9	9	0	9	7575.8517	-0.0004
7	4	3	6	3	3	7650.7130	0.0035
7	4	4	6	3	3	7650.7130	0.0370
7	4	4	6	3	4	7651.6907	-0.0058
7	4	3	6	3	4	7651.6907	-0.0392
12	1	12	11	1	11	7668.8508	0.0039
10	2	8	9	1	8	7686.4347	-0.0030
12	0	12	11	0	11	7691.6455	0.0024
12	5	8	11	5	7	7822.7163	0.0190
12	5	7	11	5	6	7822.7163	-0.0156
12	3	10	11	3	9	7826.5884	-0.0309
12	4	9	11	4	8	7826.5884	0.0526
12	4	8	11	4	7	7827.8106	-0.0005
12	3	9	11	3	8	7849.0624	-0.0055
12	1	11	11	1	10	7873.3590	0.0009
6	5	1	5	4	1	7884.0923	0.0005
6	5	1	5	4	2	7884.0923	-0.0014
6	5	2	5	4	2	7884.0923	-0.0013
6	5	2	5	4	1	7884.0923	0.0005
12	2	10	11	2	9	7908.6861	-0.0012

Table S6. Measured frequencies and residuals (in MHz) of the rotational transitions of FBEN2.

J’	K'_{-1}	K'_{+1}	J’’	K''_{-1}	K''_{+1}	ν_{obs}	ν_{obs}- ν_{calc}
4	1	4	3	1	3	2367.2713	-0.0016
3	1	2	2	0	2	2390.9920	0.0010
4	0	4	3	0	3	2409.2449	0.0007
4	2	3	3	2	2	2417.4680	-0.0012
4	3	1	3	3	0	2420.0025	-0.0277
4	2	2	3	2	1	2426.3972	0.0015
4	1	3	3	1	2	2465.4637	-0.0022
2	2	0	1	1	0	2707.5816	-0.0017
2	2	1	1	1	1	2731.2628	-0.0001
5	1	5	4	1	4	2957.5331	0.0007
5	0	5	4	0	4	3005.0705	0.0072
5	2	4	4	2	3	3020.7113	0.0064
5	4	2	4	4	1	3024.6943	-0.0031
5	4	1	4	4	0	3024.6943	-0.0062
5	3	3	4	3	2	3025.5751	-0.0086
5	3	2	4	3	1	3026.0127	-0.0026
5	2	3	4	2	2	3038.3449	0.0000
4	1	3	3	0	3	3046.4169	0.0003
5	1	4	4	1	3	3079.9992	0.0038
3	2	1	2	1	1	3291.1388	0.0022
3	2	2	2	1	2	3360.3836	0.0001
6	1	6	5	1	5	3546.8595	0.0001
6	0	6	5	0	5	3596.9950	0.0013
6	2	5	5	2	4	3623.1832	-0.0057
6	5	1	5	5	0	3629.4705	-0.0015
6	5	2	5	5	1	3629.4705	-0.0014
6	4	3	5	4	2	3630.2919	0.0102
6	4	2	5	4	1	3630.2919	-0.0039
6	3	4	5	3	3	3631.6274	0.0063
6	3	3	5	3	2	3632.7656	-0.0025
6	2	4	5	2	3	3653.4217	0.0037
6	1	5	5	1	4	3693.1848	0.0005
5	1	4	4	0	4	3717.1686	0.0007
4	2	2	3	1	2	3867.6052	0.0001
4	2	3	3	1	3	4001.6431	0.0003
7	1	7	6	1	6	4135.1613	-0.0061
7	0	7	6	0	6	4184.8993	0.0002
7	2	6	6	2	5	4224.7729	-0.0032
7	5	2	6	5	1	4234.9339	-0.0046
7	5	3	6	5	2	4234.9339	-0.0042
7	3	5	6	3	4	4237.9975	0.0068
7	3	4	6	3	3	4240.5616	0.0043
7	1	6	6	1	5	4304.6639	0.0148
3	3	0	2	2	0	4329.7825	0.0072
3	3	1	2	2	1	4330.6427	-0.0119
6	1	5	5	0	5	4405.2896	0.0007

5	2	3	4	1	3	4440.4831	-0.0010
5	2	4	4	1	4	4655.0757	0.0009
8	1	8	7	1	7	4722.4218	0.0071
8	0	8	7	0	7	4769.0859	-0.0011
8	2	7	7	2	6	4825.3322	0.0054
8	5	3	7	5	2	4840.6631	0.0014
8	5	4	7	5	3	4840.6631	0.0028
8	4	5	7	4	4	4842.5500	0.0175
8	4	4	7	4	3	4842.6417	-0.0193
8	3	6	7	3	5	4844.6221	0.0023
8	3	5	7	3	4	4849.7080	-0.0012
8	2	6	7	2	5	4892.3982	0.0074
8	1	7	7	1	6	4913.9490	0.0009
4	3	1	3	2	1	4932.5838	0.0002
4	3	2	3	2	2	4936.9275	-0.0033
6	2	4	5	1	4	5013.9068	0.0001
7	1	6	6	0	6	5112.9437	-0.0006
9	1	9	8	1	8	5308.5992	-0.0045
6	2	5	5	1	5	5320.7319	0.0006
9	0	9	8	0	8	5350.2648	0.0055
9	2	8	8	2	7	5424.7111	0.0007
9	5	4	8	5	3	5446.6785	-0.0011
9	5	5	8	5	4	5446.6785	0.0037
9	2	7	8	2	6	5514.9342	0.0008
9	1	8	8	1	7	5520.5839	-0.0014
5	3	2	4	2	2	5532.2022	-0.0010
5	3	3	4	2	3	5545.0440	-0.0014
7	2	5	6	1	5	5592.3157	0.0016
8	1	7	7	0	7	5841.9900	-0.0033
10	1	10	9	1	9	5893.7726	-0.0035
10	0	10	9	0	9	5929.3323	-0.0006
4	4	0	3	3	0	5941.3571	0.0052
4	4	1	3	3	1	5941.3571	-0.0151
7	2	6	6	1	6	5998.6494	0.0014
10	2	9	9	2	8	6022.8034	-0.0052
10	1	9	9	1	8	6124.0223	-0.0046
6	3	3	5	2	3	6126.6272	0.0008
10	2	8	9	2	7	6138.1005	-0.0037
6	3	4	5	2	4	6155.9621	0.0005
8	2	6	7	1	6	6180.0567	0.0008
11	1	11	10	1	10	6478.0077	0.0023
11	0	11	10	0	10	6507.2130	0.0012
5	4	1	4	3	1	6546.0344	0.0122
5	4	2	4	3	2	6546.1464	-0.0165
9	1	8	8	0	8	6593.4862	-0.0054
11	2	10	10	2	9	6619.5098	-0.0104
11	5	6	10	5	5	6659.7414	-0.0171
11	5	7	10	5	6	6659.7414	0.0163

11	4	8	10	4	7	6664.1709	0.0010
11	3	9	10	3	8	6664.6953	-0.0132
11	4	7	10	4	6	6665.4818	-0.0023
8	2	7	7	1	7	6688.8081	0.0007
7	3	4	6	2	4	6713.7666	0.0009
11	1	10	10	1	9	6723.7389	-0.0061
11	2	9	10	2	8	6760.7683	-0.0071
7	3	5	6	2	5	6770.7632	-0.0002
9	2	7	8	1	7	6781.0378	-0.0033
6	4	2	5	3	2	7150.3213	0.0186
6	4	3	5	3	3	7150.8488	-0.0121
8	3	5	7	2	5	7291.8875	0.0043
12	1	11	11	1	10	7319.3108	0.0213
10	1	9	9	0	9	7367.2634	0.0042
8	3	6	7	2	6	7390.6109	0.0038
9	2	8	8	1	8	7391.1074	0.0043
10	2	8	9	1	8	7398.5632	0.0032
5	5	0	4	4	0	7552.4781	-0.0037
5	5	1	4	4	1	7552.4781	-0.0041
7	4	3	6	3	3	7753.7937	0.0013
7	4	4	6	3	4	7755.4512	0.0004
9	3	6	8	2	6	7860.0987	0.0023

Table S7. Predicted spectroscopic rotational parameters and relative energies for all fenchone-benzene complexes at B3LYP-D3BJ, M06-2X and MP2 level of theory with 6-311++G (d,p) basis set.

	1			2			3		
	**B3LYP**	**MP2**	**M062X**	**B3LYP**	**MP2**	**M062X**	**B3LYP**	**MP2**	**M062X**
**A(MHz)**	767.5	768.2	773.2	804.8	803.6	811.8	843.1	857.2	869.2
**B(MHz)**	342.1	354.4	353.2	323.2	332.5	334.2	308.5	316.4	316.7
**C(MHz)**	322.5	334.9	332.9	297.9	306.8	307.1	287.8	294.5	295.0
**μ_a (D)**	1.0	0.8	1.0	-0.9	-0.8	-1.0	-1.1	-1.1	1.4
**μ_b (D)**	-0.4	-0.4	-0.5	0.1	-0.2	-0.1	-1.0	-1.1	-1.1
**μ_c (D)**	2.5	-2.3	2.5	2.6	2.4	-2.5	2.3	-1.9	-2.1
**$\Delta E(cm^{-1})$**	0	0	0	180	384	212	324	539	388
**$\Delta E_{ZPC}(cm^{-1})$**	0	0	0	195	329	56	309	482	253
**D_e (kJ/mol)**	22.0	17.4	22.1	20.0	15.5	19.8	18.4	14.0	17.8
**Figure**	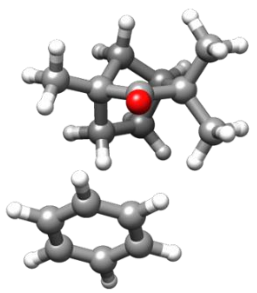			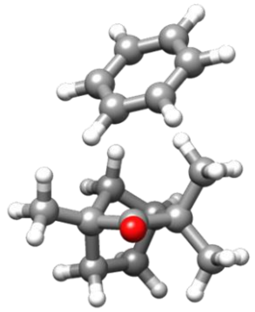			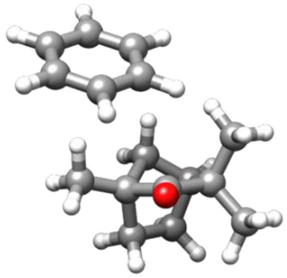		

	4			5			6		
	**B3LYP**	**MP2**	**M02X**	**B3LYP**	**MP2**	**M02X**	**B3LYP**	**MP2**	**M02X**
**A(MHz)**	885.7	868.4	890.3	871.6	863.5	877.9	744.4	742.7	747.1
**B(MHz)**	281.8	294.7	287.6	295.7	308.4	303.6	322.8	335.1	329.4
**C(MHz)**	263.9	275.7	269.2	270.9	281.8	277.1	301.1	312.9	308.8
**μ_a (D)**	-2.8	2.7	-2.8	-2.9	-2.8	2.9	0.6	0.7	0.9
**μ_b (D)**	2.1	1.9	2.2	-1.9	1.8	-1.9	-0.3	0.2	-0.2
**μ_c (D)**	0.0	-0.2	-0.0	0.4	0.2	-0.4	3.0	2.7	2.9
**$\Delta E(cm^{-1})$**	338	523	461	341	433	443	343	527	526
**$\Delta E_{ZPC}(cm^{-1})$**	316	490	243	305	380	295	313	471	305
**D_e (kJ/mol)**	18.3	14.4	17.1	18.1	14.5	17.0	18.3	14.3	16.2
**Figure**	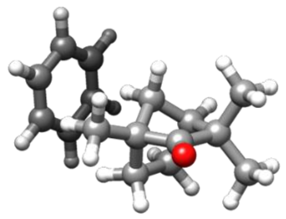			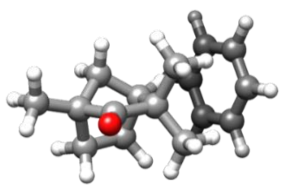			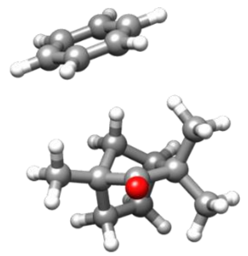		

Table S7 (cont.). Predicted spectroscopic rotational parameters and relative energies for all fenchone-benzene complexes at B3LYP-D3BJ, M06-2X and MP2 level of theory with 6-311++G (d,p) basis set.

	7			8			9		
	**B3LYP**	**MP2**	**M02X**	**B3LYP**	**MP2**	**M02X**	**B3LYP**	**MP2**	**M02X**
**A(MHz)**	945.6	951.8	959.9	795.1	804.8	798.7	773.6	781.9	789.6
**B(MHz)**	268.4	273.2	271.3	305.9	317.2	312.9	294.7	302.2	299.2
**C(MHz)**	250.9	255.1	253.2	281.6	290.5	288.8	270.0	275.7	272.9
**μ_a (D)**	-2.4	-2.1	2.3	-3.1	-3.0	3.0	-1.5	-1.6	-1.8
**μ_b (D)**	2.2	2.1	-2.2	-1.3	1.2	-1.3	-0.3	0.3	0.3
**μ_c (D)**	1.0	-1.1	1.2	-1.0	-0.7	1.2	2.8	2.5	-2.7
**ΔE(cm^{-1})**	409	723	539	412	484	530	499	788	583
**ΔE_{ZPC}(cm^{-1})**	372	625	283	367	421	439	449	676	423
**D_e (kJ/mol)**	17.9	14.0	16.3	17.3	14.0	16.0	16.6	13.1	15.7
**Figure**	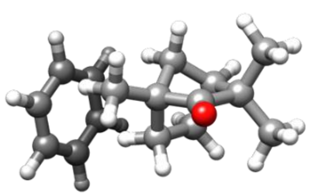			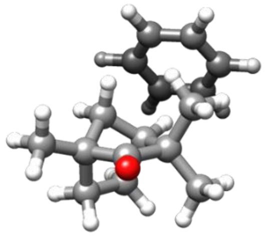			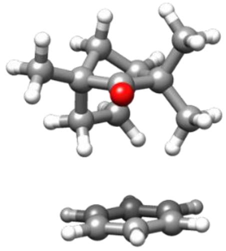		

	10			11			12		
	**B3LYP**	**MP2**	**M02X**	**B3LYP**	**MP2**	**M02X**	**B3LYP**	**MP2**	**M02X**
**A(MHz)**	772.3	782.0	789.0	956.2	965.0	971.2	998.7	917.0	958.0
**B(MHz)**	295.2	302.2	298.7	267.2	272.3	272.6	237.4	269.8	267.4
**C(MHz)**	270.6	275.7	272.6	250.4	255.3	255.5	223.9	258.5	248.6
**μ_a (D)**	1.5	-1.6	-1.9	1.9	1.7	1.9	2.3	-1.8	2.0
**μ_b (D)**	0.3	0.3	-0.3	-2.2	2.0	2.2	1.6	1.9	-1.6
**μ_c (D)**	2.8	2.5	2.7	-1.4	-1.3	1.4	-1.3	0.4	1.3
**ΔE(cm^{-1})**	500	788	598	514	755	573	660	1046	738
**ΔE_{ZPC}(cm^{-1})**	453	677	425	473	705	444	601	961	568
**D_e (kJ/mol)**	16.6	13.1	15.5	16.3	12.9	15.4	14.9	10.3	14.1
**Figure**	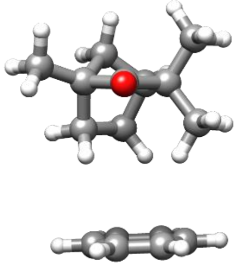			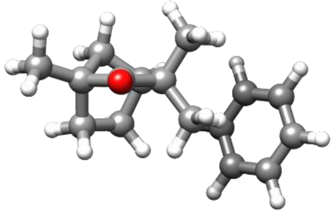			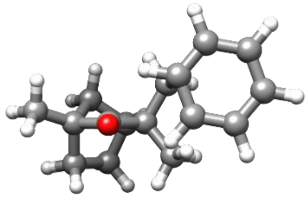		

Table S8. X, Y and Z coordinates of FPHE1 from B3LYP-D3BJ/6-311++G(d,p) geometry.

	X	Y	Z
C	0.000000	0.000000	0.000000
C	0.000000	0.000000	1.393601
C	1.218428	0.000000	2.073609
C	2.421381	0.014906	1.375185
C	2.415759	0.028746	-0.022095
C	1.197717	0.007019	-0.710644
O	3.615667	0.062993	-0.663984
O	2.912925	1.651270	-2.847697
C	2.532567	2.773906	-2.575914
C	1.372611	3.498866	-3.279821
C	1.318072	4.817949	-2.451637
C	0.800404	4.534730	-1.025231
C	1.951834	3.697431	-0.397876
C	3.073952	3.697812	-1.495145
C	2.812386	5.072185	-2.160690
C	0.101940	2.638895	-3.251541
C	1.752563	3.769234	-4.748403
C	4.469013	3.383160	-0.995328
H	2.357447	4.171120	0.498839
H	1.648130	2.687894	-0.121007
H	0.665819	5.476665	-0.488925
H	-0.157840	4.017180	-1.008443
H	0.786780	5.617387	-2.969895
H	3.425688	5.244424	-3.046601
H	2.975218	5.895655	-1.461405
H	-0.743646	3.204525	-3.652948
H	0.243553	1.750075	-3.869499
H	-0.153263	2.304800	-2.245885
H	0.919817	4.265615	-5.254730
H	2.634080	4.404663	-4.845018
H	1.957234	2.827383	-5.261453
H	5.188715	3.377230	-1.818190
H	4.786664	4.136086	-0.268843
H	4.504393	2.404901	-0.512998
H	3.498089	0.441318	-1.555194
H	-0.933989	-0.007159	1.942320
H	1.233328	-0.005902	3.157682
H	3.372939	0.028039	1.892142
H	1.198196	0.001088	-1.793362
H	-0.938627	-0.012812	-0.542666

Table S9. X, Y and Z coordinates of FPHE2 from B3LYP-D3BJ/6-311++G(d,p) geometry.

	X	Y	Z
C	0.000000	0.000000	0.000000
C	0.000000	0.000000	1.393135
C	1.219011	0.000000	2.072380
C	2.421330	0.005174	1.373912
C	2.415890	0.008080	-0.024086
C	1.197244	0.001935	-0.712384
O	3.615400	0.020465	-0.663330
O	3.215410	0.707563	-3.316642
C	3.307416	1.851683	-3.719679
C	3.704813	3.083657	-2.889568
C	3.737047	4.171445	-4.007247
C	4.925653	3.925772	-4.960827
C	4.524890	2.603513	-5.676718
C	3.077247	2.304575	-5.152531
C	2.561422	3.743483	-4.911688
C	5.029626	2.854560	-2.148168
C	2.605177	3.390800	-1.851445
C	2.263296	1.354168	-6.004093
H	4.480074	2.724557	-6.761529
H	5.208048	1.776845	-5.470348
H	5.883879	3.859643	-4.447278
H	5.000380	4.747117	-5.676819
H	3.689370	5.184093	-3.604928
H	1.583534	3.773309	-4.429291
H	2.508229	4.317194	-5.840115
H	5.369489	3.792400	-1.699268
H	4.893822	2.123251	-1.350208
H	5.818673	2.486955	-2.806037
H	2.882277	4.289460	-1.293647
H	1.629761	3.564758	-2.307985
H	2.500742	2.569380	-1.142636
H	1.261880	1.213648	-5.589443
H	2.162629	1.740595	-7.021781
H	2.740640	0.372799	-6.052527
H	3.484485	0.156138	-1.623063
H	-0.933940	-0.003195	1.941860
H	1.234626	-0.003050	3.156576
H	3.373320	0.007082	1.890873
H	1.195863	-0.006849	-1.795996
H	-0.939090	-0.006278	-0.542284

Table S10. X, Y and Z coordinates of FBEN1 from B3LYP-D3BJ/6-311++G(d,p) geometry.

	X	Y	Z
C	-0.763538	0.618187	1.384196
C	-0.941523	-0.268512	0.131085
C	0.266486	-0.030326	-0.771608
C	1.466578	-0.695723	-0.018420
C	1.750099	0.261324	1.174379
C	0.605880	1.290959	1.067564
C	0.532696	1.464006	-0.466551
C	-0.811313	-0.231962	2.659507
C	-1.899614	1.655834	1.440836
C	0.083059	-0.471940	-2.207473
O	-1.878003	-1.002208	-0.082913
H	2.316163	-0.751580	-0.703418
H	1.237497	-1.712613	0.294808
H	1.780475	-0.254329	2.133656
H	2.711300	0.765906	1.050838
H	0.770707	2.202501	1.644617
H	1.475708	1.807695	-0.899258
H	-0.267320	2.121861	-0.810836
H	-0.581167	0.382679	3.535025
H	-1.813420	-0.647226	2.786265
H	-0.110885	-1.066465	2.630292
H	-1.900080	2.324279	0.578089
H	-2.866630	1.149764	1.474149
H	-1.796647	2.266912	2.342285
H	0.981098	-0.258551	-2.794101
H	-0.111268	-1.545550	-2.254915
H	-0.763708	0.038964	-2.672839
C	0.615992	-3.950734	1.978380
C	-0.496746	-3.729944	1.168981
C	-0.410754	-3.938112	-0.206714
C	0.787577	-4.370144	-0.771476
C	1.903146	-4.587439	0.037185
C	1.817970	-4.376599	1.412433
H	0.549538	-3.786979	3.048192
H	-1.425474	-3.376387	1.598863
H	-1.272055	-3.738281	-0.831475
H	0.856081	-4.528881	-1.841756
H	2.836937	-4.917410	-0.403675
H	2.685058	-4.543275	2.041402
C	-0.763538	0.618187	1.384196

Table S11. X, Y and Z coordinates of FBEN2 from B3LYP-D3BJ/6-311++G(d,p) geometry.

	X	Y	Z
C	-0.77708	0.11317	1.12430
C	0.74817	-0.12712	1.09528
C	1.24036	0.24120	-0.30444
C	1.13450	1.80310	-0.34226
C	-0.39296	2.08165	-0.44785
C	-1.00645	0.67244	-0.31191
C	0.00160	-0.15186	-1.14335
C	-1.15100	1.05310	2.27676
C	-1.51085	-1.22555	1.32288
C	2.59826	-0.31444	-0.67554
O	1.42295	-0.51173	2.02117
H	1.67490	2.16990	-1.21848
H	1.59501	2.25913	0.53708
H	-0.75249	2.78459	0.30291
H	-0.64363	2.50022	-1.42539
H	-2.04957	0.60868	-0.62578
H	-0.18783	-1.22537	-1.14537
H	0.08017	0.19406	-2.17750
H	-2.21606	1.30046	2.23573
H	-0.94856	0.56164	3.23036
H	-0.58257	1.98428	2.26146
H	-1.30465	-1.94494	0.53084
H	-1.20907	-1.68191	2.26638
H	-2.59004	-1.04808	1.35242
H	2.59311	-1.40609	-0.67063
H	2.89396	0.02243	-1.67313
H	3.35687	0.01424	0.03896
C	-0.38064	-4.52441	-0.73450
C	0.81667	-3.85704	-0.47725
C	1.24540	-3.67185	0.83580
C	0.48056	-4.16248	1.89218
C	-0.71300	-4.83448	1.63665
C	-1.14652	-5.01229	0.32337
H	-0.71537	-4.66427	-1.75617
H	1.41151	-3.47890	-1.30090
H	2.16020	-3.13087	1.04251
H	0.81079	-4.00620	2.91225
H	-1.30953	-5.21212	2.45930
H	-2.07810	-5.52983	0.12474
C	-0.77708	0.11317	1.12430

Figure S1. Interconversion barriers between isomer 3 ↔ isomer 2 (top) and isomer 3 ↔ isomer 4 (bottom) of fenchone-phenol at the B3LYP-D3BJ/6-311++G(d,p) level of theory.

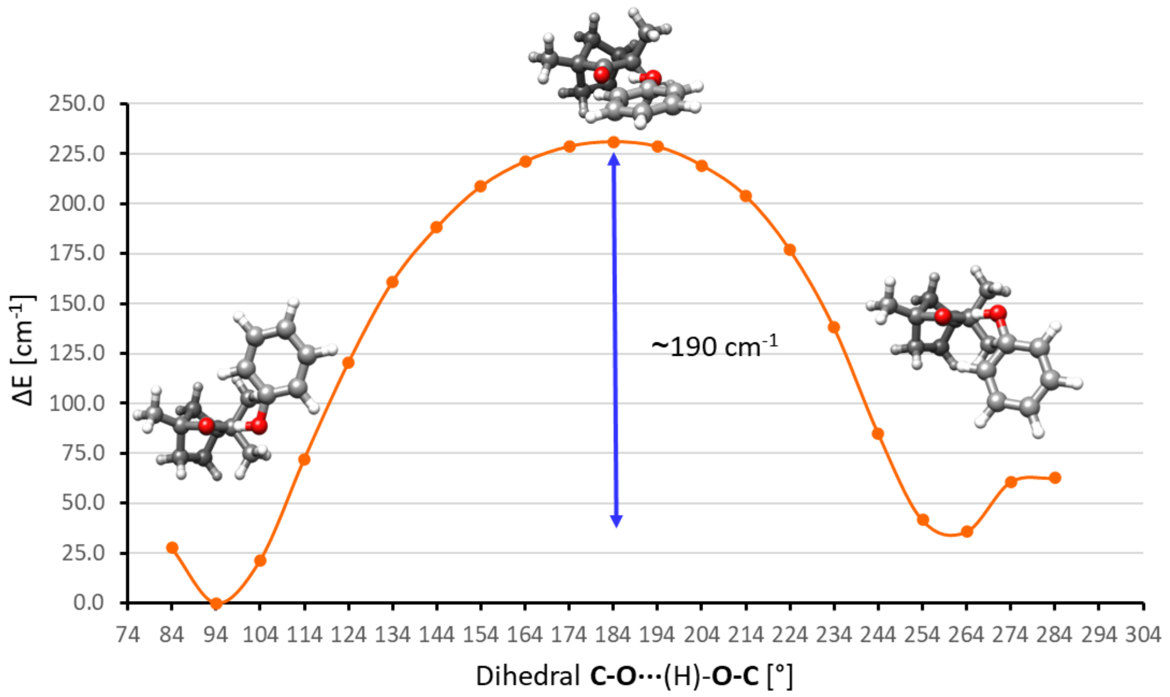

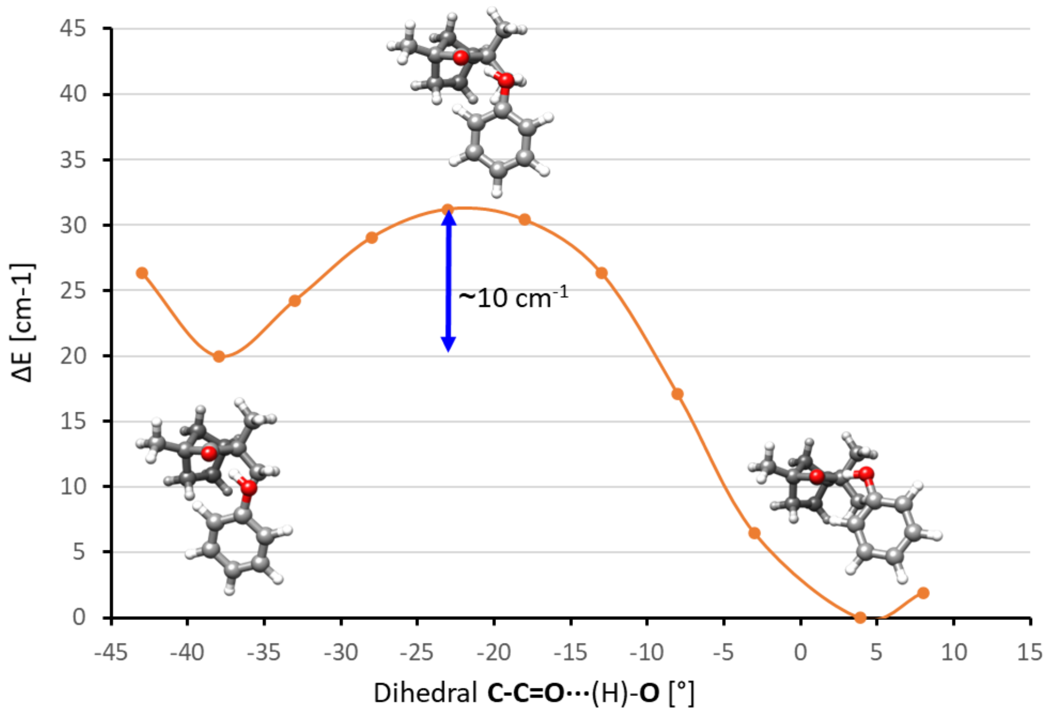

Figure S2. Sections of the broadband rotational spectra of fenchone-phenol (left) and fenchone-benzene (right). Experimental spectrum in black on top, simulated spectrum with the determined spectroscopic constants of FPHE1, FPHE2, and FBEN1, FBEN2, in different colours.

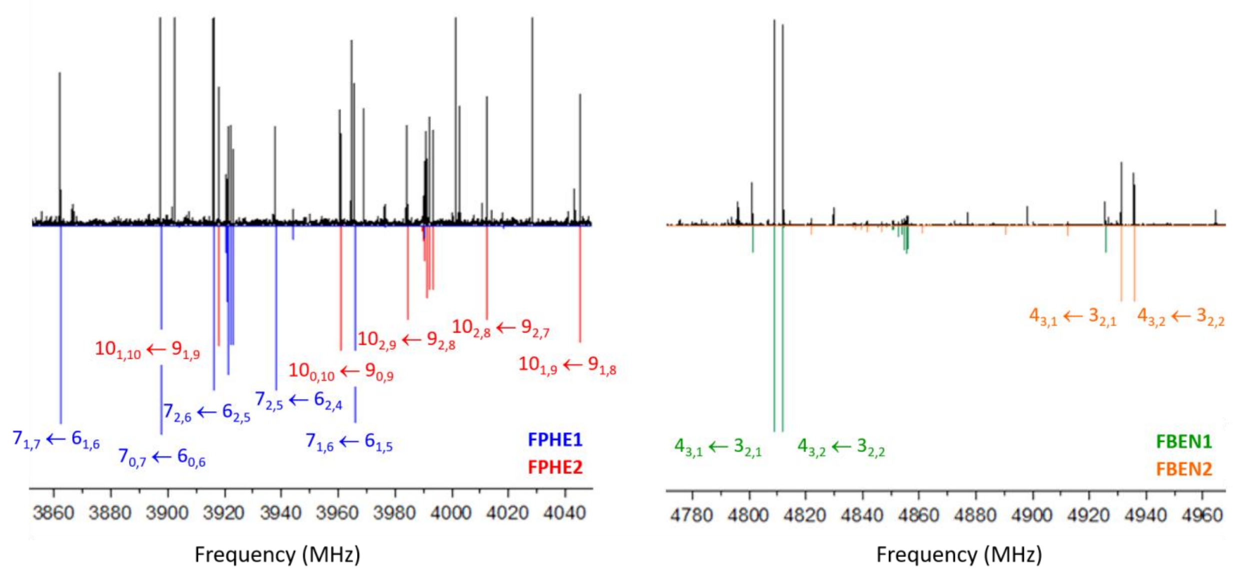

Figure S3. Comparison between the B3LYP-GD3BJ and MP2 fenchone-phenol structures (front and side views). All calculations used the 6-311++G(d,p) basis set.

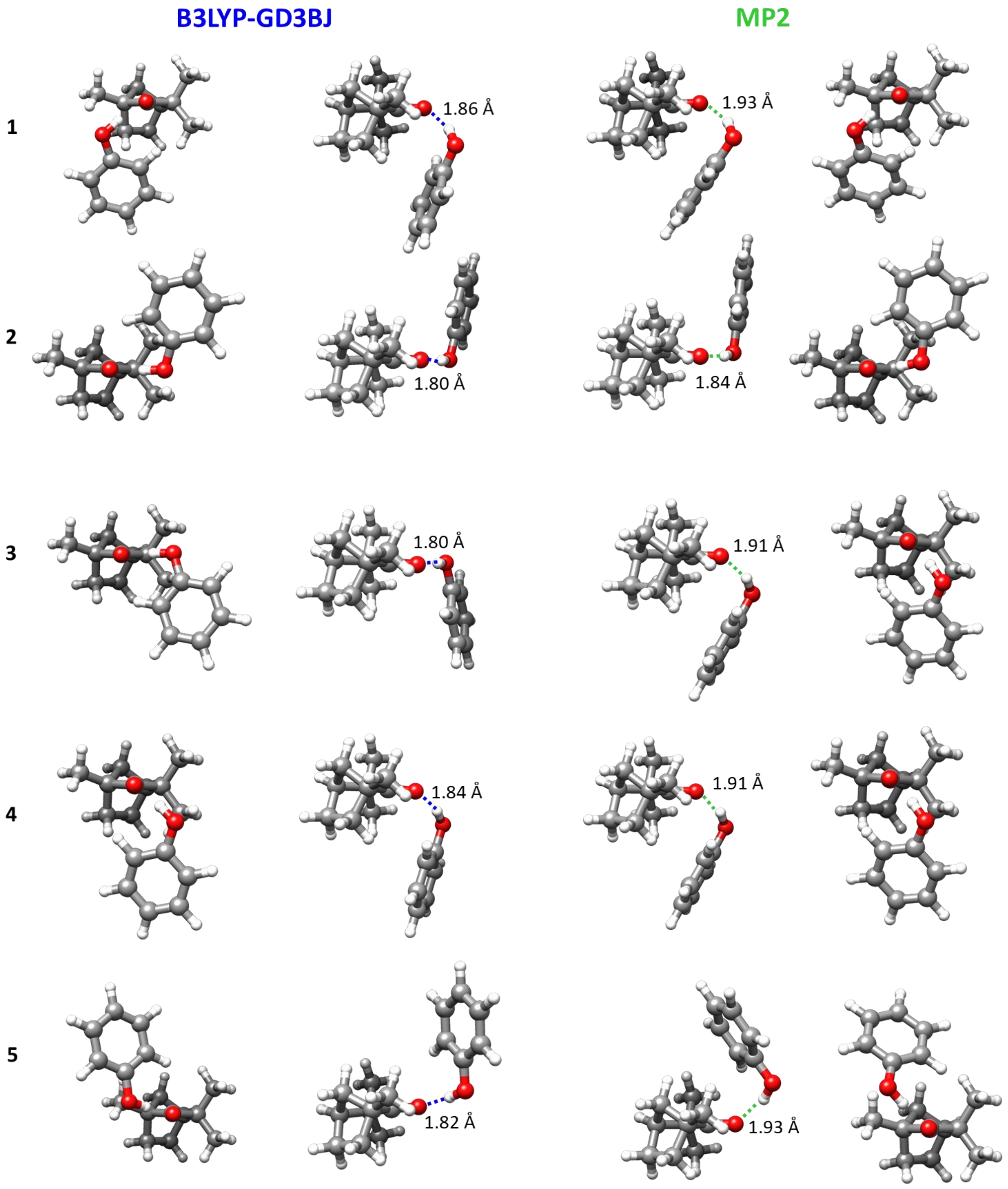

Figure S4. Comparison between the B3LYP-GD3BJ and MP2 fenchone-benzene structures (front and side views). All calculations used the 6-311++G(d,p) basis set.

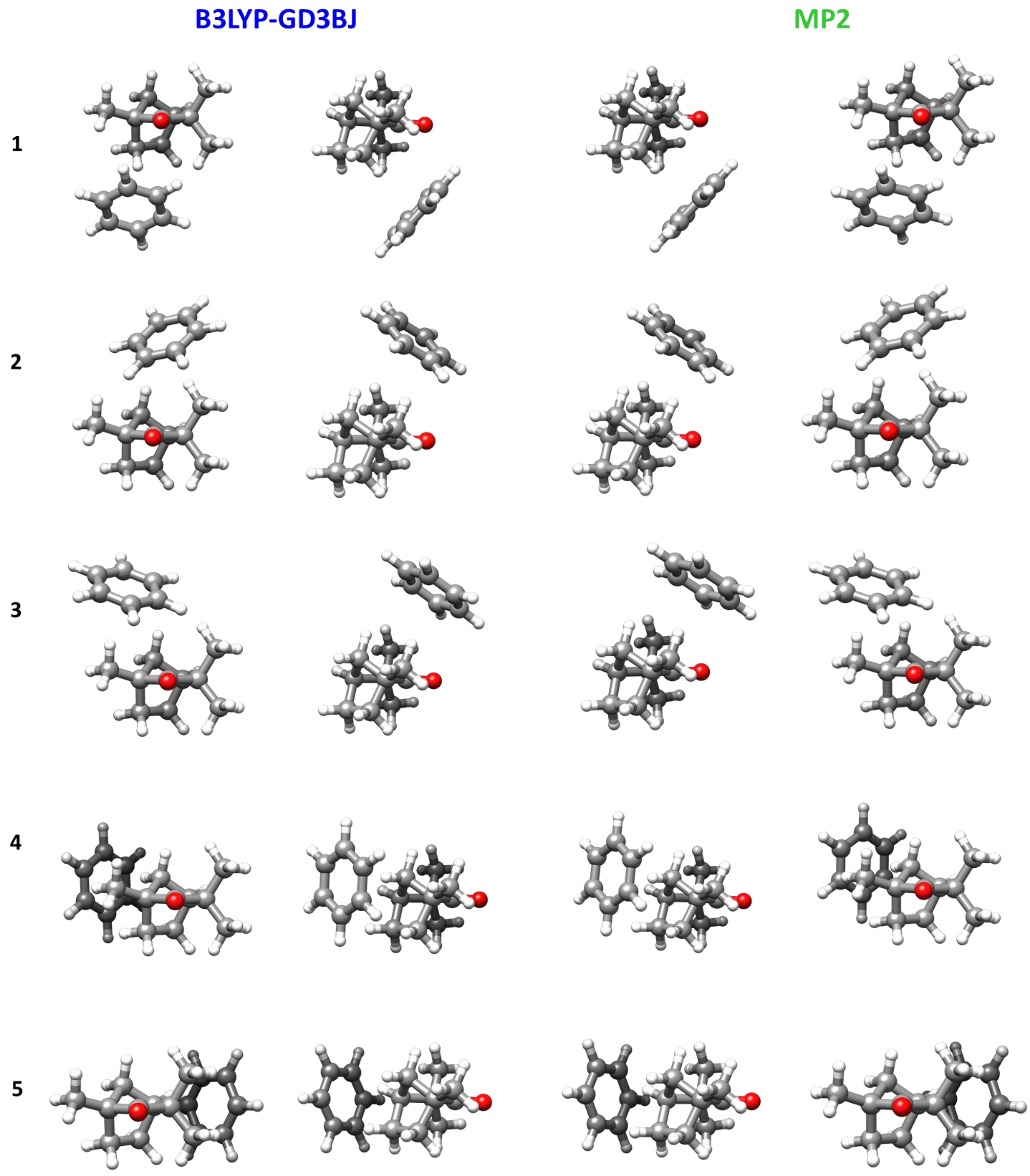

Figure S5. Comparison between the lowest-energy isomers of the fenchone-water, fenchone-ethanol, fenchone-phenol and fenchone-benzene complexes.

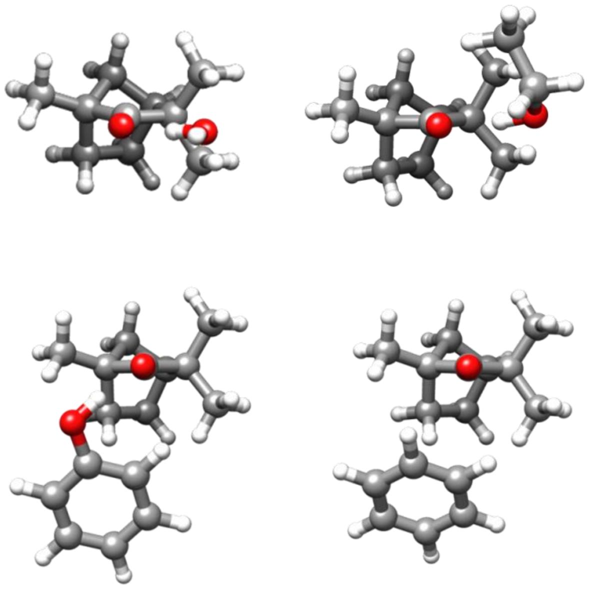

Supplement: Supplementary file 1 — Supplementary [file CHEM-26-11327-s001.pdf]
